# Chikungunya virus persists in joint-associated macrophages and promotes chronic disease in mice

**DOI:** 10.1038/s41564-026-02303-9

**Published:** 2026-04-01

**Authors:** Kristen M. Zarrella, Ryan M. Sheridan, Brian C. Ware, Bennett J. Davenport, Mariana O. L. da Silva, Dariia Vyshenska, Aspen U. Martin, Nick A. May, Erin R. Fish, Daniela Weiskopf, Jay R. Hesselberth, Daniel N. Streblow, Alex L. Greninger, Thomas E. Morrison

**Affiliations:** 1 Department of Immunology and Microbiology, University of Colorado School of Medicine, Aurora, CO USA; 2 RNA Bioscience Initiative, University of Colorado School of Medicine, Aurora, CO USA; 3 Instituto de Microbiologia Paulo de Goes, Universidade Federal do Rio de Janeiro, Rio de Janeiro, Brazil; 4 Virology Division, Department of Laboratory Medicine and Pathology, University of Washington Medical Center, Seattle, WA USA; 5 Department of Biochemistry and Molecular Genetics, University of Colorado School of Medicine, Aurora, CO USA; 6 Center for Vaccine Innovation, La Jolla Institute for Immunology, La Jolla, CA USA; 7 Department of Medicine, Division of Infectious Diseases and Global Public Health, University of California San Diego (UCSD), La Jolla, CA USA; 8 Vaccine and Gene Therapy Institute and Division of Pathobiology and Immunology, Oregon National Primate Research Center, Oregon Health and Science University, Beaverton, OR USA

**Keywords:** Viral pathogenesis, Chronic inflammation

## Abstract

Arthritogenic alphaviruses, including chikungunya virus (CHIKV), Mayaro virus and Ross River virus, cause long-lasting musculoskeletal pain and inflammation. However, the mechanisms driving chronic disease remain unclear. Here, we used single-cell RNA sequencing, spatial transcriptomics and flow cytometry to investigate joint-associated tissues in alphavirus-infected mice at a late stage of infection. We identified an accumulation of inflammatory macrophages in joint-associated tissues with elevated expression of inflammatory markers. These cells harbour CHIKV RNA, suggesting ongoing viral replication during chronic disease. We also identified an accumulation of CD4^+^ T cells in these tissues expressing *Ifng*, and found that depletion of CD4^+^ T cells diminished major histocompatibility complex class II expression on joint macrophages, highlighting their potential role in inflammation. Treatment with a small molecule inhibitor of CHIKV replication during chronic disease reduced viral RNA and joint inflammation. Our data suggest that macrophages harbour replicating viral RNA and contribute to the sustained joint inflammation associated with chronic alphavirus disease.

## Abstract (web summary)

Ongoing chikungunya virus replication leads to the accumulation of inflammatory macrophages and CD4^+^ T cells in joint-associated tissues. Inhibition of chikungunya virus replication during chronic disease reduced viral RNA and joint inflammation in mice.

## Main

Arthritogenic alphaviruses, including chikungunya (CHIKV), Mayaro (MAYV), o’nyong-nyong (ONNV), Ross River (RRV) and Sindbis viruses, are globally distributed RNA arboviruses that cause debilitating acute and chronic musculoskeletal disease^1,2^. Since 2004 CHIKV has re-emerged to cause outbreaks of increasing magnitude and severity in the Indian Ocean region and the Americas^3–5^. It is estimated that CHIKV causes 35 million annual infections and 2.8 billion people are at risk^6^, underscoring the ongoing challenge to public health systems.

Acute arthritogenic alphavirus disease onset occurs within 4–8 days of virus exposure, with symptoms including debilitating fever, polyarthralgia, joint swelling, myalgia, headache, nausea, fatigue and rash^7,8^. Studies in both humans and animal models, including non-human primates and mice, have shown that arthritogenic alphavirus infection can lead to myositis, tenosynovitis, synovitis, bone erosion and cartilage damage^9–15^.

Approximately 50% of patients with acute arthritogenic alphavirus disease progress to a chronic form of the disease, characterized by persistent or relapsing arthralgia and arthritis that can last years^16–18^. These chronic symptoms resemble those observed in individuals with rheumatoid arthritis (RA)^19^. Indeed, patients with chronic CHIKV arthritis and those with RA share similar synovial cytokine profiles characterized by elevated levels of IL-1β, IFNγ and TNF among others^20^. RA is a heterogeneous condition, with ~70% of cases being seropositive, defined by the presence of antigen-dependent autoimmune features such as rheumatoid factor and anti-cyclic citrullinated peptide antibodies, while ~30% are seronegative^21–23^. Notably, evidence indicates that viral infections, including alphaviruses, overlap in their immune cell responses and clinical features with seronegative RA^19,20,24,25^, providing a possible link between viral infection and idiopathic inflammatory arthritis while also suggesting that arthritogenic alphavirus disease and seropositive RA have distinct immunological mechanisms despite overlapping disease features.

Persistent CHIKV infection in joint-associated tissues has been hypothesized to contribute to chronic chikungunya disease^26^, but testing this hypothesis in human patients remains challenging. The joints of the wrists, hands and feet are the primary sites affected in patients with chronic CHIKV disease^2,22^, but these sites are difficult to evaluate for the presence of infectious virus, viral RNA or viral antigen. In rare examples, CHIKV antigen and RNA have been detected in synovial and muscle tissue biopsies collected from patients during the chronic phase of disease^27,28^. However, other studies failed to detect viral RNA in synovial fluid or tissue biopsies collected from patients diagnosed with chronic chikungunya^25,29^. More evidence for arthritogenic alphavirus persistence comes from experiments in animal models. In immunocompetent mice, CHIKV, MAYV, o’nyong-nyong virus and RRV RNA, along with CHIKV antigen, persist in joint-associated tissues for weeks to months following infection^14,30–38^, with fibroblasts identified as one potential cellular reservoir^37^. In CHIKV-infected macaques, joint, muscle, liver and lymphoid tissues harbour infectious CHIKV, CHIKV RNA or CHIKV antigen for weeks after inoculation^10,13^. CHIKV antigen-positive macrophages have been identified in non-human primates^13^, and macrophages have also been implicated in the pathogenesis of RA, highlighting the role of pro-inflammatory macrophages as drivers of rheumatic disease progression. However, the role CHIKV RNA^+^ fibroblasts and macrophages have in disease progression remains unclear, and the role of viral persistence, or the persistence of viral products such as antigen and RNA, in the development of chronic disease is not understood.

Here we used single-cell RNA sequencing (scRNA-seq), spatial transcriptomics and flow cytometry to investigate joint-associated tissues in alphavirus-infected mice at a late stage of infection. Our analyses identified macrophages as having the greatest transcriptional changes with CHIKV infection and as a reservoir for persistent CHIKV RNA. Similar results were observed in MAYV- and RRV-infected mice, implicating macrophages as pathogenic effectors of chronic alphavirus joint disease and sites of alphavirus persistence. Treatment of mice with a small molecule inhibitor of CHIKV replication during chronic disease implicated viral RNA replication as a driver of chronic joint inflammation. These studies provide insight into the immunopathologic mechanisms underlying chronic arthritogenic alphavirus disease.

## Results

### Joint-associated tissue inflammation during the chronic phase of CHIKV infection

**Fig. 1:**
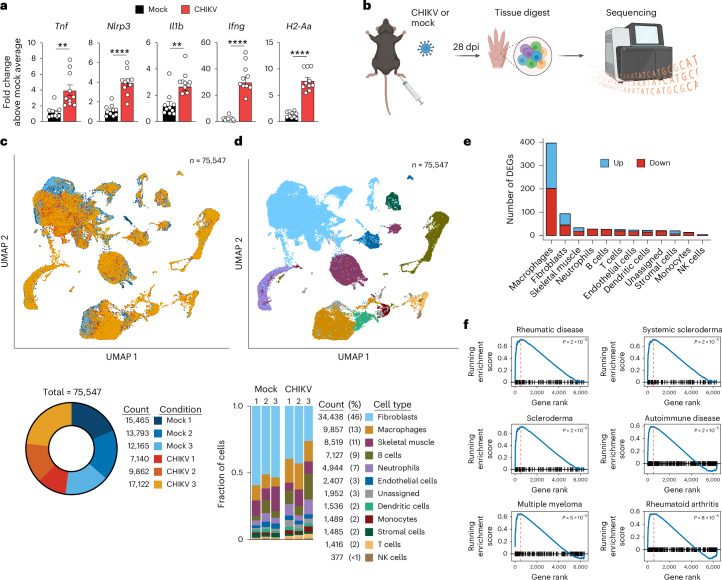
Single-cell transcriptomics analysis of joint tissue during the chronic stage of CHIKV infection. **a**, WT C57BL/6 mice were inoculated with PBS (mock; *n* = 10) or 10^3^ PFU CHIKV (*n* = 10) in the left rear footpad. At 28 dpi, inflammatory gene expression in ankle joints was quantified by RT-qPCR. Data are presented as mean values ± s.e.m. Data are from two independent experiments. *P* values were determined by two-sided unpaired Student’s *t*-test. **P < 0.01, ****P < 0.0001. **b**, Schematic of the experimental design. WT C57BL/6 mice were inoculated with PBS (mock; *n* = 3) or 10^3^ PFU CHIKV (*n* = 3) in the left rear footpad. At 28 dpi, ankle joint-associated single cells were analysed by scRNA-seq. **c**, UMAP and pie chart showing condition identity (CHIKV, red colours; mock, blue colours) and cell counts for three replicates. **d**, UMAP and bar graph showing cell type annotations for each sample. **e**, Bar graphs showing the number of genes upregulated and downregulated in CHIKV-infected mice compared with mock-infected mice (blue, upregulated; red, downregulated). **f**, GSEA was performed for disease ontology terms. Genes were ranked based on fold change in expression for macrophages from CHIKV-infected mice (red dashed line indicates the peak enrichment score; blue line indicates the running enrichment score; black lines indicate the position of gene set members in the ranked list). The top six terms are shown. *P* values were calculated using the clusterProfiler R package and corrected for multiple comparisons using the Benjamini–Hochberg procedure. Schematic in **b** created in BioRender; Morrison, T. https://biorender.com/exw31q0 (2026). Source data

To elucidate mechanisms of chronic CHIKV disease, we used a wild-type (WT) C57BL/6 mouse model of CHIKV infection that recapitulates many of the hallmarks of arthritic disease observed in patients infected with CHIKV^14^. Initially, we evaluated joint-associated tissue for signs of inflammation during the chronic stage (that is, 28 days post-infection (dpi)). This time point was selected based on previous studies^35^ showing immune-mediated clearance of an attenuated vaccine strain of CHIKV, but not WT CHIKV strains, from joint-associated tissues by 28 dpi. Previous studies^39–42^ of acute CHIKV infection in mice showed elevated pro-inflammatory markers such as IL-1β, TNF, NLRP3, major histocompatibility complex class II (MHC-II) and IFNγ. Following subcutaneous virus inoculation in the footpad, the ipsilateral ankle of CHIKV-infected mice at the chronic stage of infection also displayed an inflammatory gene expression signature characterized by heightened expression of *Tnf*, *Nlrp3*, *Il1b*, *Ifng* and *H2-Aa* (MHC-II) (Fig. 1a). NLRP3 is a component of the inflammasome involving caspase 1 or caspase 8, which cleave pro-IL-1β to the functional form, IL-1β^43^. Notably, TNF and IL-1β expression from macrophages has been implicated in cartilage and collagen destruction in RA^44,45^. Next, we analysed cells present in joint-associated tissue at 28 dpi using scRNA-seq (Fig. 1b). In total, 75,547 cells were recovered from ipsilateral ankle tissue of mock and CHIKV-infected mice (*n* = 3 mice per group) (Fig. 1c). Comparable to previous flow cytometry results^46,47^, both haematopoietic (35%) and connective tissue (62%) cells constituted the cell populations captured from the ipsilateral ankle (Fig. 1d). Macrophages (13%) and B cells (9%) constituted the largest proportions of haematopoietic cells among all cells and were accompanied by neutrophils (7%), dendritic cells (2%), monocytes (2%), T cells (2%) and natural killer (NK) cells (<1%). Macrophages and fibroblasts showed the largest number of differentially expressed genes (DEGs) compared with cells from control mice (Fig. 1e). Using gene set enrichment analysis (GSEA) to compare these scRNA-seq data with publicly available gene lists, we found that genes upregulated in joint-associated macrophages from CHIKV-infected mice showed substantial overlap with rheumatological conditions including RA and scleroderma (Fig. 1f).

### Macrophages harbour CHIKV RNA at the chronic stage of infection

**Fig. 2:**
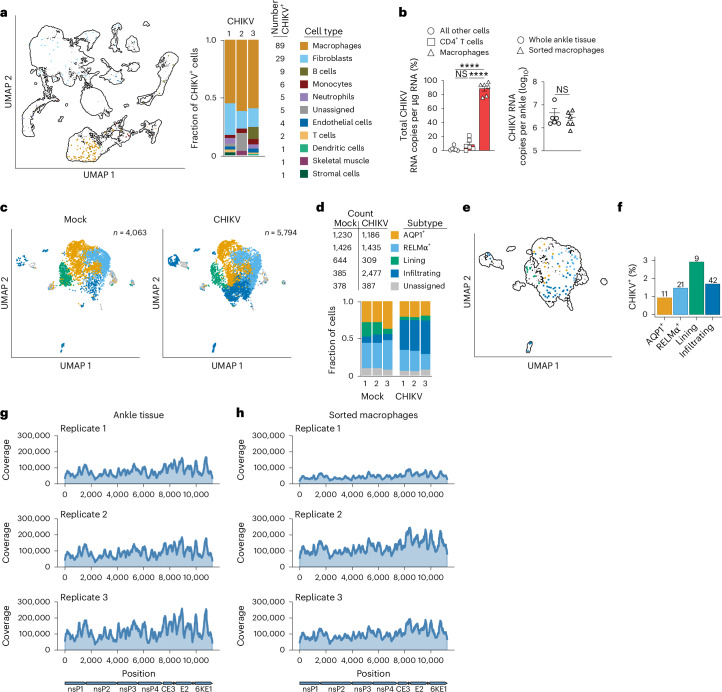
Macrophages harbour CHIKV RNA at the chronic stage of infection. **a**, UMAP and bar graph showing cells identified as CHIKV RNA^+^. **b**, WT C57BL/6 mice were inoculated with PBS (mock; *n* = 6) or 10^3^ PFU CHIKV (*n* = 6) in the left rear footpad. At 28 dpi, ankle joint-associated single cells were isolated, stained for flow cytometry (live, singlets, CD45, B220, Ly6G, NK1.1, CD11b, CD11c, CD90.2 and CD4), fixed, sorted and CHIKV RNA was quantified by RT-qPCR. Graphs show the per cent of total CHIKV RNA copies detected within sorted populations and total CHIKV RNA copies detected in sorted macrophages and whole ankle tissue. Data are from two independent experiments. Data are presented as mean values ± s.e.m. *P* values were determined by two-sided one-way ANOVA with Tukey’s multiple comparisons test or two-sided unpaired Student’s *t*-test. ****P < 0.0001. **c**, UMAP showing macrophage subsets in mock- and CHIKV-infected mice. **d**, Bar graph showing the macrophage subsets that comprise the total macrophage population. **e**, UMAP showing CHIKV RNA^+^ macrophages per subset. **f**, Frequency of CHIKV RNA^+^ macrophages in each subset. The numbers above each bar indicate the total number of CHIKV RNA^+^ macrophages detected for each subset. **g**,**h**, Coverage maps from RNA isolated from whole joint-associated tissue (**g**) or sorted joint-associated macrophages (**h**) from CHIKV-infected mice at 28 dpi (blue shading indicates the coverage at each position in the CHIKV genome). NS, not significant. Source data

**Extended Data Fig. 1:**
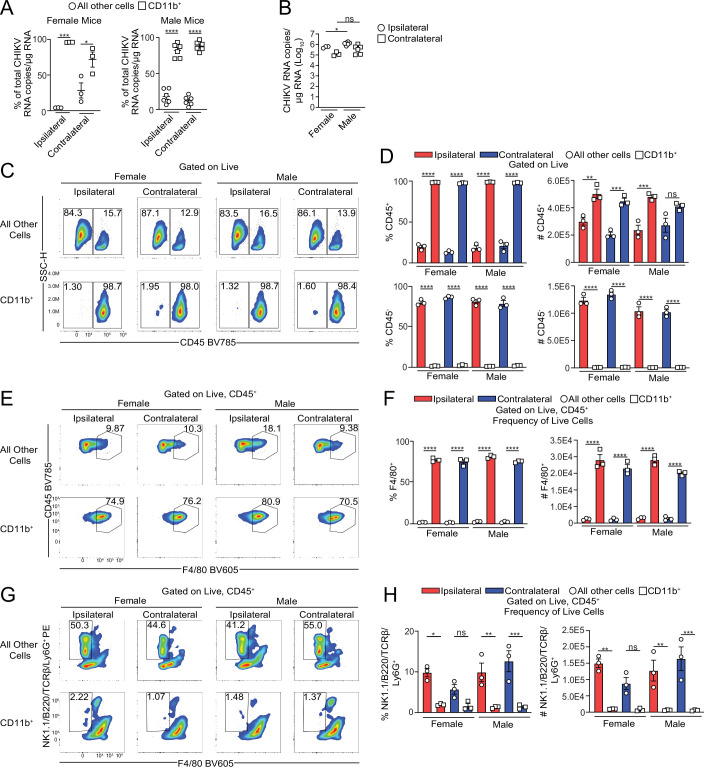
Macrophages are the major CHIKV reservoir across sexes and in both the ipsilateral and contralateral ankle joints. (**A**–**H**) WT C57BL/6 male (n = 12) or female mice (n = 6) were inoculated with PBS (mock; n = 9) or 10^3^ PFU CHIKV (n = 9) in the left rear footpad. At 28 dpi, ipsilateral and contralateral ankle joint-associated tissues were collected and CD11b^+^ cells were isolated using magnetic beads. The flow-through cells were collected as all other cells. (**A**) CHIKV RNA copies quantified by RT-qPCR. Graphs show the percent of total CHIKV RNA copies detected within each cell population. (**B**) Total CHIKV RNA copies detected in cell fractions from the ipsilateral and contralateral ankles of male and female mice. (**C**–**H**) Cell purity from the isolated cell fractions was determined by flow cytometry. (**C**) Representative flow cytometry plots of CD45^+^ and CD45^−^ cells among live, singlet cells. (**D**) Frequency and number of CD45^+^ and CD45^−^ cells among live, singlet cells. (**E**) Representative flow cytometry plots of F4/80^+^ cells among live, singlet, CD45^+^ cells. (**F**) Frequency and number of F4/80^+^ cells among live, singlet, CD45^+^ cells. (**G**) Representative flow cytometry plots of NK1.1^+^B220^+^TCRβ^+^Ly6G^+^ cells among live, singlet, CD45^+^F4/80^−^ cells. (**H**) Frequency and number of NK1.1^+^B220^+^TCRβ^+^ or Ly6G^+^ cells among live, singlet, CD45^+^F4/80^−^ cells. Data from male mice are from 2 independent experiments while data from female mice are from 1 experiment. Data are presented as mean values ± SEM. *P* values were determined by two-sided unpaired Student’s t test (**A**) or two-sided one-way ANOVA with Tukey’s multiple comparisons test (**B**–**H**). *, *P* < 0.05; ***, *P* < 0.001; ****, *P* < 0.0001. Source data

We and others reported that CHIKV RNA and antigen persist in joint-associated tissues for weeks to months after infection of immunocompetent mice, including in fibroblasts^14,31,33–35,37,48,49^. In addition, CHIKV antigen was detected in synovial tissue macrophages from a patient diagnosed with chronic CHIKV disease and in splenic macrophages months after experimental CHIKV infection of non-human primates^13,27^. Several studies^39,50–52^ found that macrophages can support alphavirus replication in vitro, in some cases for weeks, suggesting that macrophages could serve as a viral reservoir. We first evaluated whether our single-cell suspensions of ipsilateral ankle tissue captured CHIKV RNA^+^ cells. We detected similar levels of CHIKV RNA in total RNA extracted from homogenates of intact ipsilateral ankle tissue and single-cell suspensions generated by enzymatic digestion of ipsilateral ankle tissue (Supplementary Fig. 1a). Among all cells from joint-associated tissue of CHIKV-infected mice, 152 had at least 1 detected CHIKV RNA copy (0.2% of total cells), with macrophages (59%) and fibroblasts (19%) accounting for the majority (Fig. 2a). To confirm that CHIKV RNA is in macrophages at 28 dpi, we used fluorescence-activated cell sorting (FACS) of paraformaldehyde (PFA)-fixed samples to specifically isolate macrophages, CD4^+^ T cells and all other cells (Supplementary Fig. 1b). Total RNA was isolated from sorted cell populations (Supplementary Fig. 1c) and CHIKV RNA was quantified by reverse transcription quantitative PCR (RT-qPCR). The sorted macrophages contained nearly 90% of the CHIKV RNA signal detected, with the remaining ~10% spread between T cells and all other cells in the tissue. The signal detected in the macrophage population was comparable to digested whole ankle tissue (Fig. 2b). In addition, macrophages were the primary harbourers of CHIKV RNA in ipsilateral ankle joint-associated tissue of female mice and the contralateral ankle joint of both male and female mice (Extended Data Fig. 1a).

**Extended Data Fig. 2:**
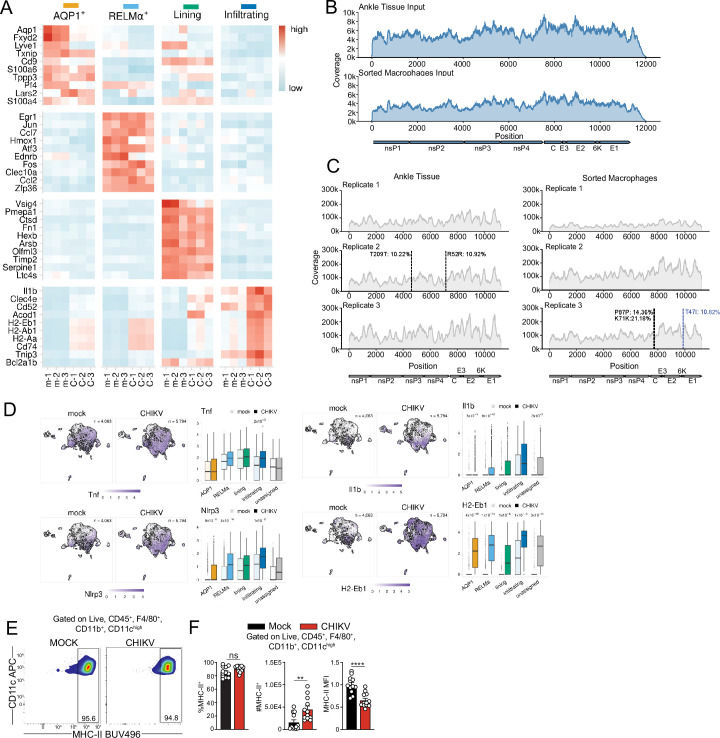
Characterization of joint macrophages during chronic CHIKV disease. (**A**) WT C57BL/6 mice were inoculated with PBS (mock; n = 3) or 10^3^ PFU CHIKV (n = 3) in the left rear footpad. At 28 dpi, ankle joint-associated single cells were analyzed by scRNA-seq. Shown are heatmaps of the top 10 marker genes for each macrophage subset. (**B**) Coverage maps for the virus inoculum used for Fig. 2g, h. (**C**) Coverage maps represented in Fig. 2g, h with synonymous (black) or nonsynonymous mutations (blue) above 10% allele frequency indicated. (**D**) UMAPs and box plots showing expression of selected inflammatory genes in macrophage subsets of mock-and CHIKV-infected mice. The center line, box limits, whiskers, and points represent the median, interquartile range (IQR), the range within 1.5xIQR from the box limits, and points outside this range (outliers), respectively. *P* values were calculated using the Wilcoxon rank sum test with Benjamini-Hochberg correction. *P* values are shown for genes that are significantly upregulated for CHIKV-infected mice. (**E**) WT C57BL/6 mice were inoculated with PBS (mock; n = 12) or 10^3^ PFU CHIKV (n = 12) in the left rear footpad. At 28 dpi, ankle joint-associated single cells were analyzed by flow cytometry. Representative flow cytometry plots for MHC-II expression on live, singlet, CD45^+^CD11b^+^CD11c^high^ cells. (**F**) Frequency, number, and gMFI of MHC-II on CD11c^high^ cells. Data are representative of 2 independent experiments. Data are presented as mean values ± SEM. *P* values were determined by two-sided unpaired Student’s t test (E-F). **, *P* < 0.01; ****, *P* < 0.0001. Source data

Resident macrophages in joint-associated tissues are a diverse population of cell types that function in tissue homeostasis and resolution of inflammation, whereas infiltrating monocyte-derived macrophages promote inflammation and injury^53,54^. An scRNA-seq study^54^ of non-infectious arthritis in mice showed that macrophages in joint-associated tissue could be divided into multiple subsets including CX_3_CR1^+^ lining macrophages, two CX_3_CR1^−^ interstitial macrophage populations (AQP1^+^ and RELMα^+^) and infiltrating macrophages characterized by expression of *Ccr2* and *Ly6c2*. Among the joint tissue-associated cells we analysed by scRNA-seq, we identified similar macrophage subsets in both mock and CHIKV-infected mice (Fig. 2c,d and Extended Data Fig. 2a). A similar cell count was evident among lining, unassigned, AQP1^+^ and RELMα^+^ macrophage subsets in mock and CHIKV-infected mice (Fig. 2d). In contrast, the number of infiltrating macrophages was ~6.5-fold higher (*P* = 9.0 × 10^−9^) in joint-associated tissue of CHIKV-infected mice (Fig. 2c,d). Among the macrophage subsets, the CHIKV RNA^+^ frequency ranged from 1% to 3% (Fig. 2e,f).

Next, we performed deep sequencing analysis of the CHIKV RNA detected in joint-associated tissue (Fig. 2g) and sorted joint macrophages (Fig. 2h). Across all replicates, we detected deep, even coverage across the full length of the CHIKV RNA genome, suggesting that the complete viral genome is maintained in cells. Analysis of CHIKV alleles that changed by >10% in frequency between CHIKV sequences recovered from tissues and macrophages with the viral sequence present in the inoculum (Extended Data Fig. 2b) revealed 2 synonymous mutations in 1 of 3 replicates from joint-associated tissue (nsP2 T209T, 10.2%; nsP4 R521R, 10.9%) and 2 synonymous (capsid P87P 14.4%; capsid K71K 21.2%) and 1 non-synonymous (6K T47I, 10.8%) mutation in 1 of 3 replicates from macrophages (Extended Data Fig. 2c), suggesting that adaptive mutations are not essential for persistence of the CHIKV genome in macrophages.

### Macrophages have an inflammatory transcriptional programme and elevated MHC-II

**Fig. 3:**
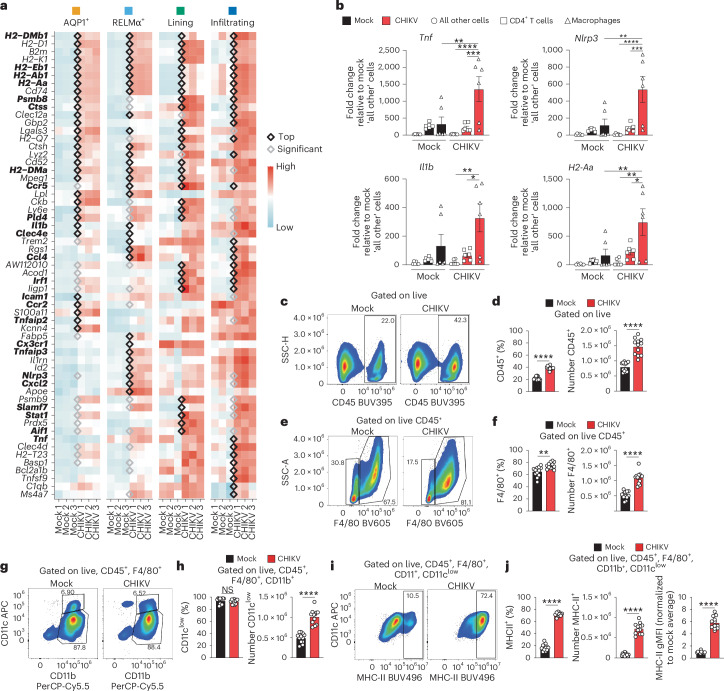
Joint tissue macrophages from CHIKV-infected mice display an activated and pro-inflammatory transcriptional programme. **a**, Heat map showing genes upregulated in macrophage subsets from CHIKV-infected mice at 28 dpi. Genes with a black diamond are significantly upregulated and are within the top 30 upregulated genes for the subset. Genes with a grey diamond are significantly upregulated in that macrophage subset but are not within the top 30 upregulated genes. Genes were first ordered based on the number of subsets sharing the upregulated gene, and then based on significance for the cell type upregulating the gene. Genes of interest are indicated in bold font. **b**, RT-qPCR analysis of inflammatory gene expression in macrophages, CD4^+^ T cells and all other cells sorted from ankle joints of mock- (*n* = 6) or CHIKV-infected (*n* = 6) mice at 28 dpi. **c**–**j**, WT C57BL/6 mice were inoculated with PBS (mock; *n* = 12) or 10^3^ PFU CHIKV (*n* = 12) in the left rear footpad. At 28 dpi, ankle joint cells were analysed by flow cytometry. **c**, Representative flow cytometry plots of CD45^+^ cells among live, singlet cells. **d**, Frequency and number of CD45^+^ cells. **e**, Representative flow cytometry plots of F4/80^+^ cells among live, singlet CD45^+^ cells. **f**, Frequency and number of F4/80^+^ cells among live, singlet CD45^+^ cells. **g**, Representative flow cytometry plots for CD11c^+^ and CD11b^+^ cells among live, singlet, CD45^+^, F4/80^+^ cells. **h**, Frequency and number of CD11c^low^ cells. **i**, Representative flow cytometry plots of MHC-II expression on macrophages. **j**, Frequency and number of MHC-II^+^ macrophages, and MHC-II gMFI. Data are representative of two independent experiments. Data are presented as mean values ± s.e.m. *P* values were determined by two-sided one-way ANOVA with Tukey’s multiple comparisons test (**b**) or two-sided unpaired Students’ *t*-test (**d**, **f**, **h** and **j**). *P < 0.05, **P < 0.01, ***P < 0.001, ****P < 0.0001. Source data

Analysis of gene expression among all macrophage subsets revealed that those in joint-associated tissue of CHIKV-infected mice showed elevated expression of numerous genes involved in an inflammatory response, including *Ctss*, *Pld4*, *Clec4e*, *Ccl4*, *Tnfaip2*, *Tnf*, *Tnfaip3*, *Slamf7*, *Aif1*, *Cxcl2*, *Tnf*, *Il1b* and *Nlrp3*^55–58^ (Fig. 3a), and many genes associated with immune cell infiltration, such as *Ccr5*, *Ccr2* and *Cx3cr1* (ref. ^59^). Analysis of gene expression patterns across the different macrophage subsets revealed some differences in the top DEGs (Fig. 3a). Macrophages in each subset expressed elevated levels of MHC-II-associated genes, such as *H2-Dmb1*, *H2-Eb1*, *H2-Ab1*, *H2-Aa* and *H2-Dma*, and other genes regulated by IFNγ, such as *Psmb8*, *Irf1*, *Icam1*, *Stat1*, *Slamf7*, *Nlrp3* and *Tnf*^60–62^. Taken together, these data suggest that macrophages within the joint tissue are composed of heterogenous subsets that are pro-inflammatory and MHC-II^+^ in CHIKV-infected mice, even at 4 weeks post virus inoculation. Joint tissue fibroblasts in CHIKV-infected mice also showed elevated expression of inflammatory genes, although to a lesser extent compared with macrophages (Supplementary Fig. 2).

To confirm these findings, macrophages, CD4^+^ T cells and all other cells were sorted by FACS from the joint tissue of control and CHIKV-infected mice at 28 dpi, and gene expression in sorted cells was measured by RT-qPCR. Consistent with our scRNA-seq analysis, macrophages from CHIKV-infected mice showed increased expression of *Tnf*, *Nlrp3*, *Il1b* and *H2-Aa* compared with CD4^+^ T cells and all other cells (Fig. 3b and Extended Data Fig. 2d). However, only *Tnf*, *Nlrp3* and *H2-Aa* were elevated relative to joint macrophages sorted from uninfected control mice. Remarkably, all other cells isolated from the joint tissue of CHIKV-infected mice expressed relatively low levels of these inflammatory genes (Fig. 3b), suggesting that macrophages are a primary driver of joint tissue inflammation during the chronic stage of CHIKV infection.

**Extended Data Fig. 3:**
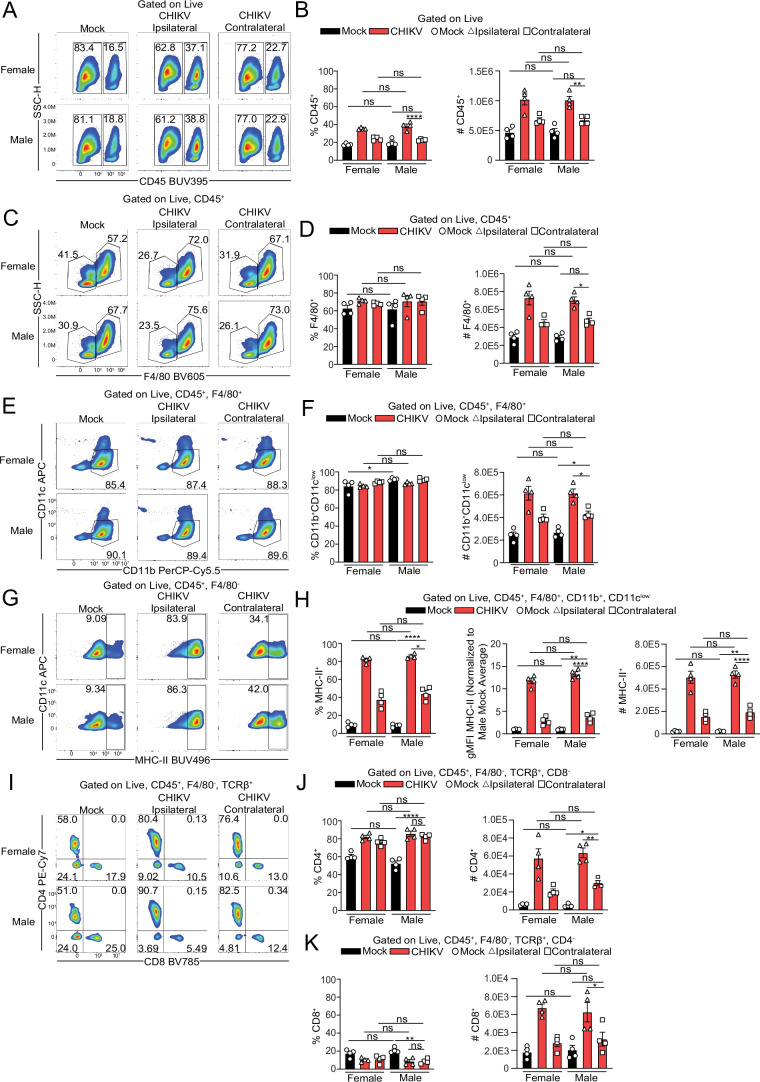
Elevated MHC-II^+^ macrophages in the contralateral ankle during chronic CHIKV disease. (**A**–**K**) WT C57BL/6 male (n = 4) or female mice (n = 4) were inoculated with PBS (mock; n = 8) or 10^3^ PFU CHIKV (n = 8) in the left rear footpad. At 28 dpi, ipsilateral and contralateral ankle joint-associated single cells were analyzed by flow cytometry. (**A**) Representative flow cytometry plots of CD45^+^ and CD45^−^ cells among live, singlet cells. (**B**) Frequency and number of CD45^+^ cells. (**C**) Representative flow cytometry plots of F4/80^+^ cells among live, singlet, CD45^+^ cells. (**D**) Frequency and number of F4/80^+^ cells among live, singlets, CD45^+^ cells. (**E**) Representative flow cytometry plots for CD11c^+^ and CD11b^+^ cells among live, singlet, CD45^+^, F4/80^+^ cells. (**F**) Frequency and number of CD11c^low^ cells. (**G**) Representative flow cytometry plots of MHC-II expression on macrophages. (**H**) Frequency and number of MHC-II^+^ macrophages, and MHC-II geometric mean fluorescent intensity (gMFI). (**I**) Representative flow cytometry plots of CD4^+^ and CD8^+^ cells among live, singlet, CD45^+^F4/80^−^TCRβ^+^ cells. (**J**, **K**) Frequency and total number of CD4^+^ and CD8^+^ cells among live, singlet, CD45^+^F4/80^−^TCRβ^+^ cells. Data are from 1 experiment. Data are presented as mean values ± SEM. *P* values were determined by two-sided one-way ANOVA with Tukey’s multiple comparisons test (**A**–**K**). *, *P* < 0.05; **, *P* < 0.01; ****, *P* < 0.0001. Source data

We further evaluated macrophages present in joint-associated tissues at 28 dpi using flow cytometry. There was an increase in CD45^+^ (Fig. 3c,d) and F4/80^+^ (Fig. 3e,f) cells in CHIKV-infected tissue compared with controls. Among F4/80^+^ cells, we found a heterogenous population of CD11b- and CD11c-expressing cells (Fig. 3g). The majority (>80%) were CD11b^+^, CD11c^low^ (Fig. 3g,h). We evaluated these populations for surface expression of MHC-II (Fig. 3i,j and Extended Data Fig. 2e,f) and observed the F4/80^+^CD11b^+^CD11c^high^ population expressed similar levels of MHC-II in both mock- and CHIKV-infected mice (Extended Data Fig. 2e,f). In contrast, the F4/80^+^CD11b^+^CD11c^low^ cells in CHIKV-infected mice showed a fourfold increase in the total number of cells expressing MHC-II, and a sixfold increase in the surface expression of MHC-II (geometric mean fluorescence intensity (gMFI)) compared with the same cells from uninfected mice (Fig. 3i,j). MHC-II^+^ macrophages were also elevated in the joint-associated tissue of female mice and contralateral ankle joint tissue of both male and female mice (Extended Data Fig. 3). Therefore, there is a sustained population of MHC-II^+^ macrophages in joint-associated tissue of CHIKV-infected mice during chronic disease.

### CD4^+^ T cells regulate macrophage MHC-II expression during chronic CHIKV disease

**Fig. 4:**
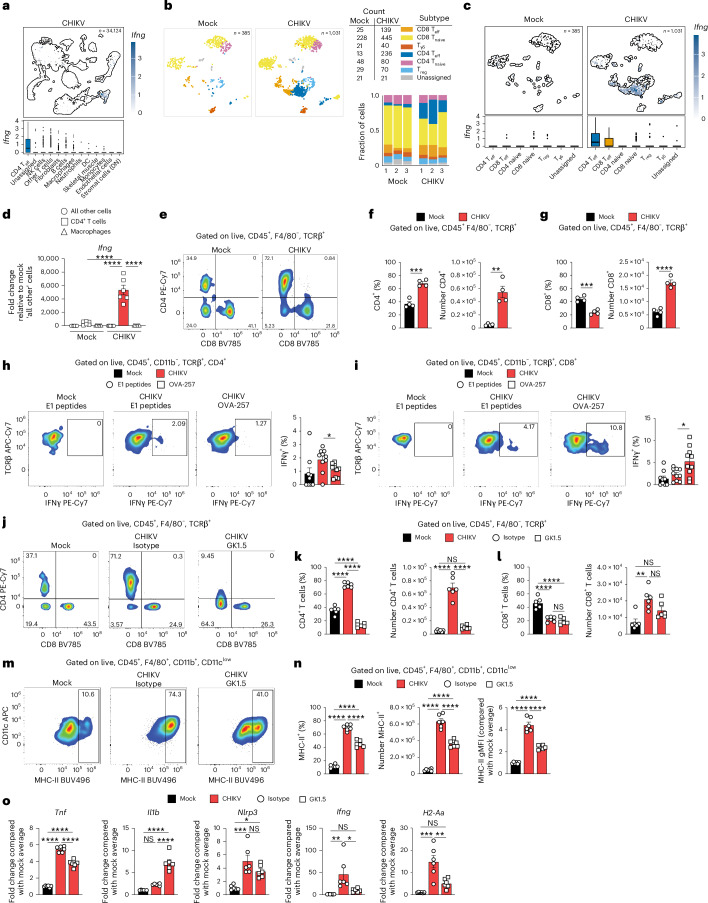
CD4^+^ T cells regulate MHC-II^+^ macrophages during chronic CHIKV joint disease. **a**, UMAP and box plots comparing *Ifng* expression for CD4 T_eff_ cells and all other cell types. The centre line, box limits, whiskers and points represent the median, interquartile range (IQR), the range within 1.5× IQR from the box limits and points outside this range (outliers), respectively. **b**, UMAP showing T cell subsets. The table and bar graph show the total counts and fraction for each T cell subset, respectively. **c**, UMAP and box plots showing *Ifng* expression for T cell subsets from mock- and CHIKV-infected mice. The centre line, box limits, whiskers and points represent the median, IQR, the range within 1.5× IQR from the box limits and points outside this range (outliers), respectively. **d**, RT-qPCR analysis of *Ifng* expression in macrophages, CD4^+^ T cells and all other cells sorted from the ankle joints of mock- or CHIKV-infected mice at 28 dpi. **e**–**g**, WT C57BL/6 mice were inoculated with PBS (mock; *n* = 4) or 10^3^ PFU CHIKV (*n* = 4) in the left rear footpad. At 28 dpi, ankle joint cells were analysed by flow cytometry. **e**, Representative flow cytometry plots of CD4^+^ and CD8^+^ cells among live, singlet CD45^+^F4/80^−^TCRβ^+^ cells. **f**,**g**, Frequency and total number of CD4^+^ (**f**) and CD8^+^ (**g**) cells among live, singlet CD45^+^F4/80^−^TCRβ^+^ cells. **h**,**i**, WT C57BL/6 mice were inoculated with PBS (*n* = 6) or 10^3^ PFU CHIKV-OVA (*n* = 6) in the left rear footpad. At 28 dpi, ankle joint single-cell suspensions were stimulated ex vivo with the indicated peptides for 5 hours in the presence of brefeldin A. **h**, Representative flow cytometry plots of IFNγ^+^ cells among live, singlet CD45^+^CD11b^−^TCRβ^+^CD4^+^ cells. Bar graph shows the frequency of IFNγ^+^ cells in each group. **i**, Representative flow cytometry plots of IFNγ^+^ cells among live, singlet CD45^+^CD11b^−^TCRβ^+^CD8^+^ cells. Bar graph shows the frequency of IFNγ^+^ cells in each group. **j**–**o**, WT C57BL/6 mice were inoculated with PBS (*n* = 9) or 10^3^ PFU CHIKV (*n* = 10) in the left rear footpad. At 14 dpi, mice were injected intraperitoneally with 250 μg of anti-mouse CD4 antibody (clone GK1.5) or an isotype control antibody every 5 days for 15 days. At 28 dpi, ankle joint-associated tissue was analysed by RT-qPCR or flow cytometry. **j**, Representative flow cytometry plots of CD4^+^ and CD8^+^ cells among live, singlet CD45^+^F4/80^−^TCRβ^+^ cells. **k**,**l**, Frequency and number of CD4^+^ and CD8^+^ T cells. **m**, Representative flow cytometry plots depicting CD11c^low^ and MHC-II^+^ cells among live, singlet CD45^+^F4/80^+^CD11b^+^CD11c^low^ cells. **n**, Frequency, number and gMFI of MHC-II^+^ cells among live, singlet CD45^+^F4/80^+^CD11b^+^CD11c^low^ cells. **o**, RT-qPCR analysis of gene expression. Data are normalized to GAPDH mRNA levels and are expressed as the relative expression (*n*-fold increase) over expression in mock-inoculated mice. Data are representative of two to three independent experiments. Data are presented as mean values ± s.e.m. *P* values were determined by two-sided one-way ANOVA with Tukey’s multiple comparison test (**d**, **j**–**o**) or two-sided unpaired Student’s *t*-test (**e**–**i**). *P < 0.05, **P < 0.01, ***P < 0.001, ****P < 0.0001. DC, dendritic cell; DN, double negative. Source data

**Extended Data Fig. 4:**
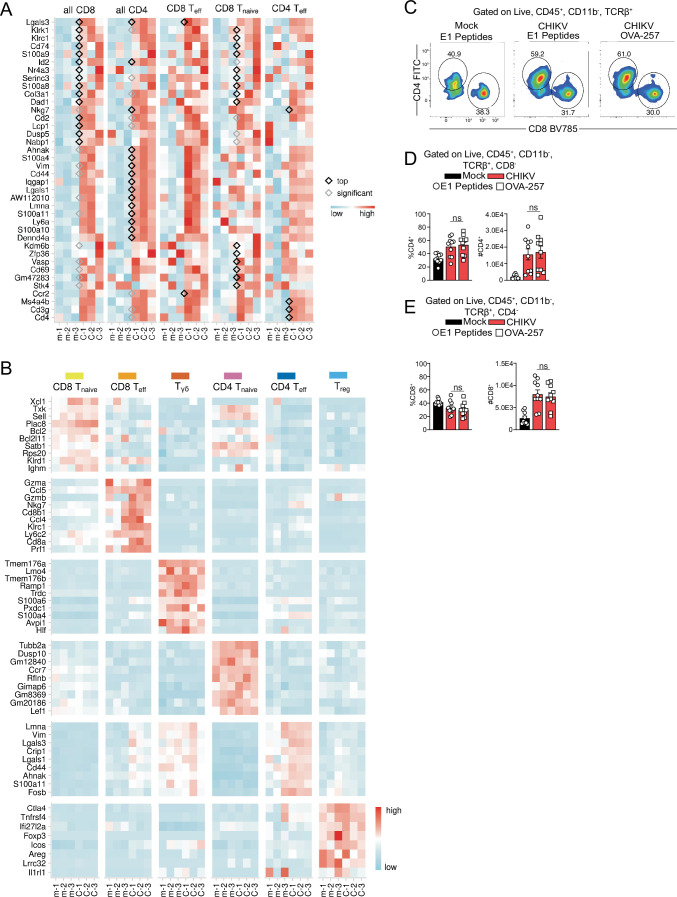
scRNA-seq analysis of joint-associated T cells during chronic CHIKV disease. (**A**) Heatmap showing genes upregulated in T cell subsets from CHIKV-infected mice at 28 dpi (m = mock; C = CHIKV). Genes with a grey diamond are significantly upregulated. Genes with a black diamond are significantly upregulated and are within the top 15 upregulated genes for the subset. Genes were first ordered based on the number of subsets sharing the upregulated gene, then based on significance for the cell type upregulating the gene. Mean expression values were scaled separately for each subset. (**B**) Heatmap showing the expression of the top 10 marker genes for each T cell subset (m= mock; C = CHIKV). ScRNA-seq data are representative from one experiment with 3 mice/group. (**C**–**E**) WT C57BL/6 mice were inoculated with PBS (mock; n = 9) or 10^3^ PFU CHIKV-OVA (n = 10) in the left rear footpad. At 28 dpi, ankle joint single cell suspensions were stimulated *ex vivo* with the indicated peptides for 5 h in the presence of brefeldin A. (**C**) Representative flow cytometry plots of CD4^+^ and CD8^+^ cells among live, singlet, CD45^+^F4/80^−^TCRβ^+^ cells. (**D**) Frequency and number of CD4^+^ T cells in stimulation groups. (**E**) Frequency and number of CD8^+^ T cells in stimulation groups. Data are presented as mean values ± SEM. *P* values were determined by two-sided unpaired Student’s t test (**D**, **E**). Source data

MHC-II expression by macrophages can be induced by IFNγ and can, in turn, promote IFNγ production by CD4^+^ T cells^63^. In our scRNA-seq dataset, elevated expression of *Ifng* was detected predominantly within CD4^+^ T cells (Fig. 4a–c). Next, we analysed the top genes expressed in CD4^+^ and CD8^+^ T cells (Extended Data Fig. 4a) and then further divided *Cd3e*-expressing cells into clusters of naive and effector (T_eff_) CD4^+^ and CD8^+^ T cells, regulatory (T_reg_), γδ and unassigned T cells (Fig. 4b and Extended Data Fig. 4b). Compared with uninfected control mice, the number of CD4^+^ T_eff_ (18-fold) and CD8^+^ T_eff_ (5.5-fold) cells were increased in joint-associated tissue of CHIKV-infected mice (Fig. 4b). Both CD4^+^ and CD8^+^ T_eff_ cells showed increased levels of *Ifng* transcripts compared with T_eff_ cells in uninfected control mice and other T cells in joint tissue of CHIKV-infected mice (Fig. 4c). We also evaluated *Ifng* expression in cell populations sorted from joint tissue at 28 dpi (Fig. 4d). This analysis revealed that CD4^+^ T cells express >200-fold greater *Ifng* compared with macrophages, all other cells and CD4^+^ T cells from uninfected control mice, supporting a functional interaction between joint tissue macrophages and CD4^+^ T cells during chronic CHIKV infection. Previous studies in humans reported T_reg_ cells are reduced in frequency during chronic CHIKV disease^64^, and expanding T_reg_ cells in mice reduced acute CHIKV-induced disease^65^. However, our scRNA-seq analysis did not detect any differences in the frequency of T_reg_ cells in ankle joint-associated tissue of control and CHIKV-infected mice during the chronic stage (Fig. 4b). By flow cytometry, the frequency and number of CD4^+^ T cells in CHIKV-infected mice was more than six times greater than that in uninfected control mice (Fig. 4e,f). The number of CD8^+^ T cells, but not the frequency, was also increased in joint tissue from CHIKV-infected mice (Fig. 4g).

Patients with chronic CHIKV disease show circulating CHIKV-specific CD4^+^ T cell responses, primarily targeting viral E1 and nsP1 proteins^66^. To assess the local CD4^+^ T cell response, we isolated joint-associated cells at 28 dpi from uninfected control mice and mice infected with a recombinant CHIKV strain encoding a CD8^+^ T cell receptor epitope from ovalbumin (OVA)^67^. Stimulation with overlapping CHIKV E1 peptides triggered responses in CD4^+^ T cells, confirming antigen specificity (Fig. 4h), while CD8^+^ T cells responded only to the OVA-257 epitope (Fig. 4i). There was no effect on the frequency or number of CD4^+^ or CD8^+^ T cells after restimulation (Extended Data Fig. 4c–e). These data suggest that either persistent CHIKV antigen presentation is maintained or that antigen-specific CD4^+^ and CD8^+^ T cells are maintained in joint tissue independent of antigen presentation.

**Extended Data Fig. 5:**
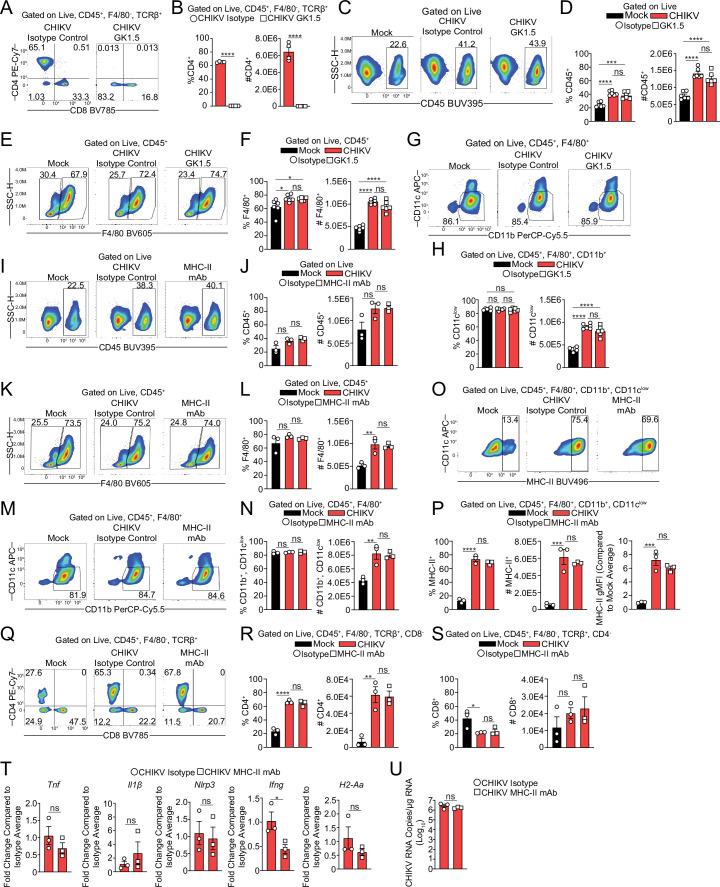
CD4^+^ T cells regulate MHC-II^+^ macrophages during chronic disease. (**A**–**H**) WT C57BL/6 mice were inoculated with PBS (mock; n = 6) or 10^3^ PFU CHIKV (n = 6) in the left rear footpad. At 14 dpi, mice were injected intraperitoneally with 250 μg of anti-mouse CD4 antibody (clone: GK1.5) or an isotype control antibody every 5 days. At 28 dpi, ankle joint-associated tissue and the spleen were analyzed by flow cytometry. (**A**) Representative flow cytometry plots of splenic CD4^+^ and CD8^+^ cells among live, singlet, CD45^+^F4/80^−^TCRβ^+^ cells. (**B**) Frequency and number of splenic CD4^+^ T cells. (**C**) Representative flow cytometry plots of CD45^+^ cells in joint-associated tissue among live, singlet cells. (**D**) Frequency and number of CD45^+^ cells among live, singlet cells. (**E**) Representative flow cytometry plots of F4/80^+^ cells in joint-associated tissue among live, singlet, CD45^+^ cells. (**F**) Frequency and number of F4/80^+^ cells in joint-associated tissue among live, singlet, CD45^+^ cells. (**G**) Representative flow cytometry plots of CD11b^+^ and CD11c^+^ cells in joint-associated tissue among live, singlet, CD45^+^, F4/80^+^ cells. (**H**) Frequency and number of CD11c^low^ cells in joint-associated tissue among live, singlets, CD45^+^, F4/80^+^, CD11b^+^ cells. (**I**–**U**) WT C57BL/6 mice were inoculated with PBS (mock; n = 3) or 10^3^ PFU CHIKV (n = 6) in the left rear footpad. At 14 dpi, mice were injected intraperitoneally with 200 μg of anti-mouse MHC-II antibody or an isotype control antibody every 3 days. At 28 dpi, ankle joint-associated tissue was analyzed by flow cytometry or RT-qPCR. (**I**) Representative flow cytometry plots of CD45^+^ cells among live, singlet cells. (**J**) Frequency and number of CD45^+^ cells. (**K**) Representative flow cytometry plots of F4/80^+^ cells among live, singlet, CD45^+^ cells. (**L**) Frequency and number of F4/80^+^ cells among live, singlets, CD45^+^ cells. (**M**) Representative flow cytometry plots for CD11c^+^ and CD11b^+^ cells among live, singlet, CD45^+^F4/80^+^ cells. (**N**) Frequency and number of CD11c^low^ cells. (**O**) Representative flow cytometry plots of MHC-II expression on macrophages. (**P**) Frequency and number of MHC-II^+^ macrophages, and MHC-II geometric mean fluorescent intensity (gMFI). (**Q**) Representative flow cytometry plots of CD4^+^ and CD8^+^ cells among live, singlet, CD45^+^F4/80^−^TCRβ^+^ cells. (**R**, **S**) Frequency and total number of CD4^+^ and CD8^+^ cells among live, singlet, CD45^+^F4/80^−^TCRβ^+^ cells. (**T**) RT-qPCR analysis of gene expression. Data are normalized to GAPDH mRNA levels and are expressed as the relative expression (n-fold increase) over expression in mock-inoculated mice. (**U**) CHIKV RNA copies quantified by RT-qPCR. Data from CD4^+^ T cell depletion are representative of 2 independent experiments while data from MHC-II mAb treatment are from 1 experiment. Data are presented as mean values ± SEM. *P* values were determined by two-sided one-way ANOVA with Tukey’s multiple comparisons test (D, F, J, H, L, N, P, R, S) or two-sided unpaired Student’s t test (A, B, T, U). *, *P* < 0.05; **, *P* < 0.01; ***, *P* < 0.001; ****, *P* < 0.0001. Source data

CD4^+^ T cells contribute to joint inflammation and swelling during acute CHIKV infection of mice^33,41,49,68^, but their role in chronic disease is less understood. We found that during chronic infection, joint CD4^+^ T cells express elevated *Ifng* transcripts (Fig. 4a–d), implicating them in sustained inflammation, and our scRNA-seq analysis revealed that CD4^+^ T cells displayed a transcriptional programme suggestive of cell–cell interactions. For example, laminin (*Lmna*), a cytoskeletal protein critical for immunological synapse formation^69^, was among the top upregulated genes in CD4^+^ T cells of CHIKV-infected mice (Extended Data Fig. 4a). To investigate their role, we depleted CD4^+^ T cells starting at 14 dpi. By 28 dpi, flow cytometry confirmed effective depletion in both joint tissue and spleen, with no impact on CD8^+^ T cells or total CD45^+^ cell numbers (Fig. 4j–l and Extended Data Fig. 5a–d). While macrophage numbers remained unchanged (Extended Data Fig. 5e–h), the frequency, number and gMFI of MHC-II^+^ macrophages were reduced following CD4^+^ T cell depletion (Fig. 4m,n). Further analysis showed that depletion of CD4^+^ T cells reduced *Ifng* and *H2-Aa* (MHC-II) expression, but unexpectedly increased expression of *Il1b* (Fig. 4o). As IFNγ can suppress *Il1b* transcription^70,71^, these data suggest distinct regulatory mechanisms are involved in different aspects of chronic joint inflammation following CHIKV infection. In addition, treating CHIKV-infected mice during the chronic phase with an MHC-II-blocking antibody did not affect lymphocyte numbers (Extended Data Fig. 5i–s) or alter expression of pro-inflammatory genes or viral RNA, with the notable exception of IFNγ, which was reduced (Extended Data Fig. 5t,u). These findings support a link between MHC-II on macrophages and IFNγ expression by CD4^+^ T cells, and a reciprocal relationship in which CD4^+^ T cells regulate MHC-II presentation on macrophages.

### Joint macrophages harbour viral RNA at the chronic stage of MAYV and RRV infection

**Extended Data Fig. 6:**
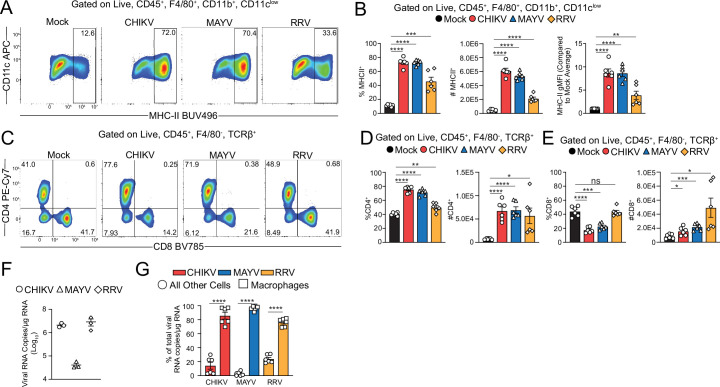
Joint macrophages harbor viral RNA during the chronic stage of MAYV and RRV infection. (**A**–**D**) WT C57BL/6 mice were inoculated with PBS (mock; n = 6) or 10^3^ PFU CHIKV (n = 6), MAYV (n = 6), or RRV (n = 6) in the left rear footpad. At 28 dpi, ankle joint-associated single cells were assessed by flow cytometry. (**A**) Representative flow cytometry plots of CD11c^low^ and MHC-II^+^ cells among live, singlet, CD45^+^F4/80^+^CD11b^+^CD11c^low^ cells. (**B**) Frequency, number, and gMFI of MHC-II^+^ cells among live, singlet, CD45^+^F4/80^+^CD11b^+^CD11c^low^ cells. (**C**) Representative flow cytometry plots of CD4^+^ and CD8^+^ cells among live, singlet, CD45^+^F4/80^−^TCRβ^+^ cells. (**D**, **E**) Frequency and number of CD4^+^ and CD8^+^ T cell populations among live, singles, CD45^+^F4/80^−^TCRβ^+^ cells. (**F**, **G**) WT C57BL/6 mice were inoculated with PBS (mock) or 10^3^ PFU CHIKV, MAYV, or RRV in the left rear footpad. At 28 dpi, ankle tissue was homogenized in Trizol (n = 3/group), or joint-associated single cells were isolated, stained for flow cytometry, fixed, sorted, and viral RNA was quantified by RT-qPCR (n = 6/group). Viral RNA copies in (**F**) whole ankle tissue or (**G**) sorted macrophages and all other cells. Data is representative of 2 experiments. Data are presented as mean values ± SEM. *P* values were determined by two-sided one-way ANOVA with Tukey’s multiple comparison test (B, D, E) or two-sided Student’s t test (G). *, *P* < 0.05; **; ***, *P* < 0.001; ****, *P* < 0.0001. Source data

Next, we evaluated if mice infected with MAYV or RRV also have elevated MHC-II^+^ macrophages and CD4^+^ T cells in joint tissue during the chronic phase, and if joint macrophages harbour MAYV and/or RRV RNA. At 28 dpi, the frequency and number of MHC-II^+^F4/80^+^CD11b^+^CD11c^low^ macrophages, and the gMFI of MHC-II on macrophages, were elevated in joint-associated tissue of MAYV- and RRV-infected mice (Extended Data Fig. 6a,b and Supplementary Fig. 3a–f). In addition, the frequency and number of CD4^+^ T cells were also increased in joint tissue of MAYV- and RRV-infected mice, as in CHIKV-infected mice (Extended Data Fig. 6c,d). Notably, while the frequency of CD8^+^ T cells in joint tissue was diminished in CHIKV- and MAYV-infected mice compared with control mice, this was not observed in RRV-infected mice. Nevertheless, all virus-infected mice showed an increased number of CD8^+^ T cells in joint tissue (Extended Data Fig. 6e). In all virus infections, the elevated number of MHC-II^+^ macrophages and T cells was associated with the presence of viral RNA in joint-associated tissue (Extended Data Fig. 6f). To determine if this persistent MAYV and RRV RNA is also in macrophages, we sorted macrophages and all other cell types from joint-associated tissue at 28 dpi (Supplementary Fig. 3g) and quantified viral RNA in each sorted cell population. In each case, viral RNA was predominantly detected in macrophages (Extended Data Fig. 6g), suggesting that these cells serve as a key reservoir for arthritogenic alphaviruses.

### Viral RNA^+^ cells are closely localized during chronic CHIKV disease

**Fig. 5:**
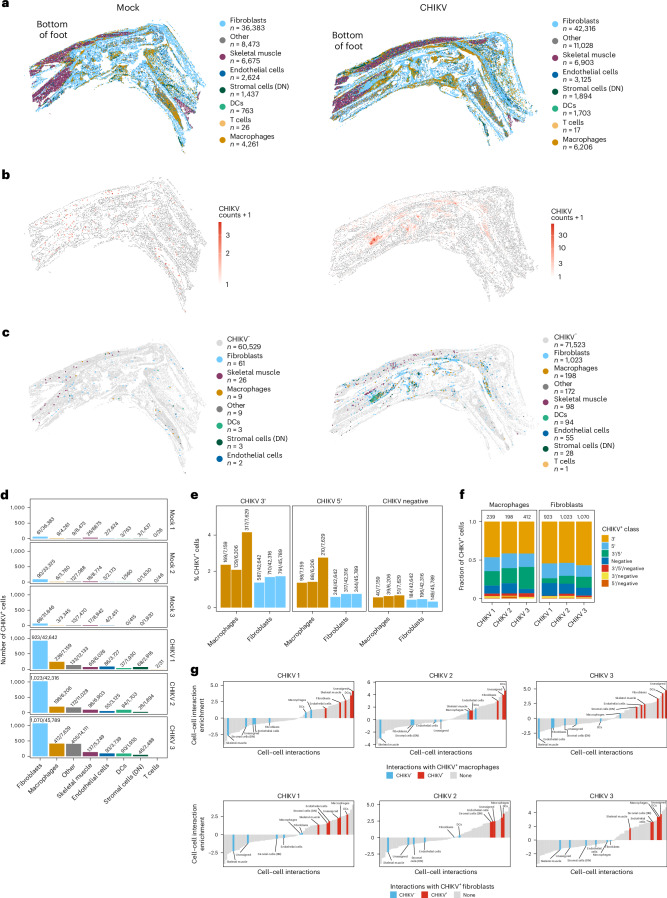
Spatial transcriptomic analysis of joint-associated tissue. WT C57BL/6 mice were inoculated with PBS (mock; *n* = 3) or 10^3^ PFU CHIKV (*n* = 3) in the left rear footpad. At 28 dpi, joint-associated tissue was analysed by spatial transcriptomics. **a**, Annotated cell types in mock- and CHIKV-infected joint-associated tissue. **b**, CHIKV RNA counts in cells of mock- and CHIKV-infected joint-associated tissue. **c**, Annotated cell types that are CHIKV RNA^+^. **d**, The number of CHIKV RNA^+^ cells per cell type in each replicate. **e**, The percentage of macrophages and fibroblasts that were positive for 3′ positive-sense RNA (3′), 5′ positive-sense RNA (5′) and negative-strand RNA (negative) CHIKV-specific probes. **f**, Bar graphs showing the fraction of CHIKV RNA^+^ macrophages and fibroblasts that were positive for 3′ RNA positive-sense (5′), 5′ RNA positive-sense (3′), negative-strand RNA (negative) or some combination of CHIKV-specific probes. **g**, Cell proximity enrichment maps where each highlighted bar represents a cell type that showed increased (positive enrichment) or decreased (negative enrichment) interactions with CHIKV RNA^+^ macrophages (top) or fibroblasts (bottom). CHIKV RNA^−^ cells are indicated in blue while CHIKV RNA^+^ cells are in red.

To determine the spatial organization of CHIKV RNA^+^ cells within joint-associated tissue during the chronic stage, we performed virus-inclusive spatial transcriptomics (Fig. 5 and Supplementary Figs. 4 and 5). We confidently annotated dendritic cells, endothelial cells, fibroblasts, macrophages, skeletal muscle cells, stromal cells and other cell types with a prediction confidence score above 0.5 (Fig. 5a and Supplementary Figs. 4 and 5a,b). In tissues from CHIKV-infected mice, we detected numerous cells that had between 1 and 150 CHIKV RNA counts (Fig. 5b and Supplementary Fig. 5c,d). The CHIKV RNA^+^ cells were predominately composed of fibroblasts and macrophages (Fig. 5c,d and Supplementary Fig. 5d). Across all replicates, we found that the CHIKV signal in both fibroblasts and macrophages was higher for probes that targeted sequences in the 3′ end of the viral genome (Fig. 5e), which are also found in the viral subgenomic mRNA produced in infected cells. In addition, we detected both positive-sense and negative-sense CHIKV RNA, in some cases in the same cells (Fig. 5e,f), further suggesting active CHIKV RNA replication. Finally, using cell proximity analysis (4), we identified a positive association between CHIKV RNA^+^ macrophages and fibroblasts and other CHIKV RNA^+^ cells, suggesting these cell types sustain CHIKV RNA in joint-associated tissue during chronic disease (Fig. 5g), possibly via cell-to-cell spread which occurs during CHIKV infection in vivo even in the presence of neutralizing antibodies^72^.

### CHIKV RNA replication promotes joint inflammation during chronic disease

**Fig. 6:**
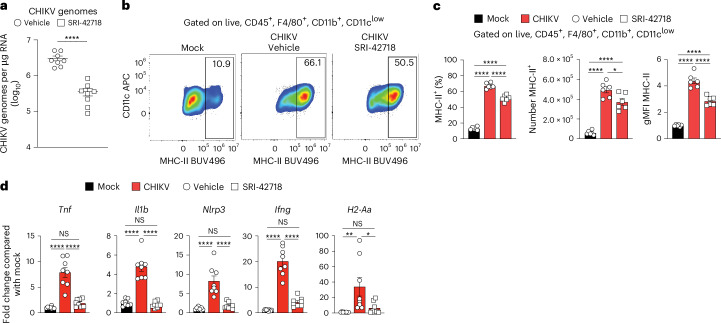
Antiviral therapy initiated during the chronic stage reduced CHIKV RNA and inflammatory gene expression. WT C57BL/6 mice were inoculated with PBS (mock; *n* = 8) or 10^3^ PFU CHIKV (*n* = 8) in the left rear footpad. At 28 dpi, mice were administered 60 mg kg^−1^ of a small molecule antiviral, SRI-42718, or vehicle by oral gavage every 12 hours for 7 days. At 35 dpi, ankle joint-associated tissue was isolated for analysis by flow cytometry and RT-qPCR. **a**, CHIKV RNA copies quantified by RT-qPCR. **b**, Representative flow cytometry plots of CD11c^low^ and MHC-II^+^ cells among live, singlet CD45^+^F4/80^−^TCRβ^+^CD11c^low^ cells. **c**, Frequency, number and gMFI of MHC-II^+^ cells among live, singlet CD45^+^F4/80^+^CD11b^+^CD11c^low^ cells. **d**, RT-qPCR analysis of gene expression. Data are normalized to GAPDH mRNA levels and are expressed as the relative expression (*n*-fold increase) over expression in mock-inoculated mice. Data are representative of two experiments. Data are presented as mean values ± s.e.m. *P* values were determined by two-sided unpaired Student’s *t*-test (**a**) or two-sided one-way ANOVA with Tukey’s multiple comparison test (**b**–**d**). *P < 0.05, **P < 0.01, ****P < 0.0001. Source data

To test if a direct-acting antiviral can reduce tissue levels of viral RNA and mitigate joint inflammation when administered during the chronic phase, we treated mice with a small molecule inhibitor of alphavirus replication. At 28 dpi, we administered vehicle only or 60 mg kg^−1^ of inhibitor every 12 hours for 7 days through oral gavage. At 35 dpi, mice treated with the inhibitor had reduced CHIKV RNA burden in joint-associated tissue compared with vehicle-treated mice (Fig. 6a), further suggesting that CHIKV RNA persists by a continuous replication mechanism. Although mice treated with the inhibitor showed little to no differences in the number or frequency of macrophages or T cells present in joint-associated tissue (Supplementary Fig. 6), inhibitor-treated mice had reduced MHC-II expression on macrophages (Fig. 6b,c). Importantly, mice treated with the inhibitor had reduced inflammatory gene expression in joint-associated tissue, similar to that detected in mock-infected control mice (Fig. 6d). These data suggest that viral RNA replication occurs during chronic disease and contributes to joint tissue inflammation.

## Discussion

Our results suggest that joint tissue macrophages and fibroblasts are cellular sites of alphavirus persistence and that macrophages are key drivers of chronic joint tissue inflammation. During the chronic phases, we found that joint macrophages and fibroblasts had the largest number of DEGs compared with cells from control mice. Indeed, all subsets of macrophages identified in the joint (that is, AQP1^+^, RELMα^+^, lining and infiltrating) showed elevated expression of numerous inflammatory genes and MHC-II-associated genes, and many of these same genes, including *Il1b*, *Tnf*, *Nlrp3* and *H2-Aa*, were identified in previous analyses of gene expression in the joint-associated tissue of CHIKV-infected mice during the acute and chronic stages^34,73^.

The numbers of macrophages in joint-associated tissues were also elevated during the chronic stage in mice infected with either MAYV or RRV. Importantly, an increased number of macrophages was also identified in synovial and muscle tissue biopsies from patients with chronic chikungunya or chronic RRV-associated arthritis^27,28,74^. These findings suggest that macrophages are critical drivers of joint inflammation during chronic alphavirus-induced arthritis. Notably, studies during the acute stage of CHIKV infection in mice identified the accumulation of MHC-II^+^ macrophages in joint-associated tissue, and depletion of these cells ameliorated acute joint pathology^39,41^. We found that day 28 joint tissue macrophages in CHIKV-infected mice expressed elevated levels of *Nlrp3* and *Il1b*. Previous transcriptional analysis^75^ of peripheral blood mononuclear cells from patients with acute CHIKV infection identified activation of the NLRP3 inflammasome as a key feature of CHIKV infection. In addition, exposure of murine bone marrow macrophages to CHIKV or MAYV induced IL-1β production by a mechanism that was dependent on NLRP3 inflammasome activation^75,76^, and inhibition of NLRP3 or *Nlrp3* deficiency reduced CHIKV- and MAYV-induced inflammation during acute infection in mice^40,76^. These data highlight that there are overlapping features of acute and chronic joint inflammation following alphavirus infection, and suggest that sustained viral activation of the NLRP3 inflammasome in joint tissue macrophages may promote chronic disease.

In addition to macrophages, CD4^+^ T cells have also been identified in synovial and muscle tissue biopsies from patients with chronic chikungunya^27,28^, and studies^33,41,49,68^ in mice implicated these cells as mediators of joint pathology during acute CHIKV infection. Recently, an analysis^66^ of human peripheral blood mononuclear cells collected years after infection from patients with chikungunya found that CHIKV-specific CD4^+^ T cell responses, particularly against epitopes in nsP1 and E1, were significantly stronger in patients with chronic disease compared with those in which symptoms had resolved. Using this same E1 peptide pool, we identified E1-specific CD4^+^ T cells in the joints of CHIKV-infected mice during the chronic stage. In addition, our scRNA-seq analysis found that the number of CD4^+^ T_eff_ cells was increased and that these cells expressed elevated *Ifng*. Depletion of CD4^+^ T cells reduced MHC-II expression on joint tissue macrophages, suggesting a functional interaction between these cells. Similar macrophage–CD4^+^ T cell interactions were observed in mice during acute CHIKV infection^41^, further highlighting that there are overlapping features of acute and chronic joint inflammation following CHIKV infection.

The role of persistent infection or the persistence of viral products in chronic alphavirus-induced arthritis in humans remains to be determined. Although a few studies^27,28,74^ have detected viral RNA and/or antigen in musculoskeletal tissues of patients with chronic alphavirus arthritis, other studies^25,29^ have reported negative results. Nevertheless, numerous studies suggest that alphaviruses are capable of establishing persistent infection. Indeed, long-term persistence of viral RNA but not infectious virus was detected in the brain of mice following Sindbis and Semliki Forest virus infection, and this viral RNA supported a resurgence of infectious virus production when immune control was blocked^77,78^. In addition, non-cytopathic, persistent alphavirus RNA replication can be observed in mammalian cells in vitro^79–82^. We identified joint tissue macrophages and fibroblasts as the predominant cellular sites of CHIKV RNA persistence, and deep sequencing analysis of this RNA detected reads spanning the full-length genome. Moreover, our spatial transcriptomics analysis revealed the presence of both positive-sense and negative-sense CHIKV RNA, with fibroblasts and macrophages containing the highest proportion of cells showing this viral RNA replication signature. These findings suggest that these cell types serve as sites of persistent viral RNA replication and their close proximity may support cell–cell viral spread. In support of this idea, therapeutic treatment of mice with a small molecule inhibitor of viral replication reduced the persistent CHIKV RNA in joint-associated tissue. The reduced viral RNA in mice that received antiviral treatment was associated with decreased MHC-II expression on macrophages and pro-inflammatory gene expression. These data provide compelling evidence that CHIKV RNA replication in macrophages and fibroblasts promotes joint tissue inflammation and supports the hypothesis that antiviral therapy could mitigate chronic disease severity.

A cell type pertinent to CHIKV pathogenesis is skeletal muscle fibres^37,83,84^, which are typically lost when preparing single-cell suspensions for analysis by flow cytometry or scRNA-seq. Thus, many of our analyses did not capture information for this cell type. Nevertheless, we did detect skeletal muscle cells by spatial transcriptomics analysis of foot and ankle tissue and found only small numbers of these cells were CHIKV RNA^+^, suggesting that skeletal muscle cells may not be a major cellular site of viral RNA persistence. However, further work is needed to confirm these findings. Our deep sequencing analysis of the CHIKV genome in joint tissue and joint tissue macrophages suggests that adaptive mutations are not required for persistence of viral RNA. Similarly, a previous RNA-sequencing study^34^ of CHIKV-infected mouse feet at day 30 did not detect significant viral sequence variants. However, each of these analyses was performed at a single time point and it remains possible that adaptive mutations would become enriched in the viral genome over longer periods of time. Finally, while the mouse model of chronic chikungunya used in this study recapitulates many of the hallmarks of chronic chikungunya in humans, the model has some limitations, including lower levels of persistent viral RNA and inflammation in joint tissues that are distal to the site of inoculation and the absence of a relapsing–remitting disease pattern observed in some patients with chronic chikungunya^22^.

## Methods

### Biosafety

All experiments were reviewed and approved by the Institutional Biosafety Committee of the University of Colorado Anschutz under protocol 1051. All work involving infectious CHIKV was conducted in approved Biosafety Level 3 (BSL-3) and animal BSL-3 laboratories.

### Viruses

CHIKV AF15561, MAYV CH (provided by Scott Weaver, University of Texas Medical Branch) and RRV T48 were generated from cDNA clones as described previously^35^. CHIKV-VENKL, which encodes the CD8^+^ TCR epitope SIINFEKL, was generated as previously described^47^. Briefly, plasmids were linearized by NotI, PacI or SacI digestion and used as a template for in vitro transcription with SP6 DNA-dependent RNA polymerase (Ambion). Capped RNAs were concentrated by lithium chloride precipitation, evaluated by gel electrophoresis and then electroporated into BHK-21 cells. At 24–28 hours post electroporation, cell culture supernatants were collected and clarified by centrifugation. Clarified supernatants were aliquoted and stored at −80 °C and virus titre was determined by plaque assay.

### Mouse experiments

WT C57BL/6J mice were obtained from The Jackson Laboratory. All experiments were performed in 4-week-old male or female mice. Mice were group housed in a facility with a 14 hours light and 10 hours dark cycle, ambient temperature of 72 ± 2 °F and ambient humidity of 40 ± 10%. No statistical methods were used to predetermine mouse sample sizes but our sample sizes are similar to those reported in previous publications^72,83,85–89^. Mice were randomly assigned to experimental groups, anaesthetised with isoflurane vapours and inoculated in the left rear footpad with a 10 μl volume containing 10^3^ plaque-forming units (PFU) of virus diluted in phosphate buffered saline (PBS) with 1% foetal bovine serum (FBS) using a Hamilton syringe and 30 G needle. Following euthanasia, blood was collected and mice were intracardially perfused with 10 ml of PBS at the indicated time points. To deplete CD4^+^ T cells, mice were intraperitoneally inoculated with 250 μg of either isotype control antibody (LTF-2; Bioxcell) or anti-CD4 antibody (GK1.5; Bioxcell) every 5 days starting at 14 dpi. Mice were killed at 28 dpi. Depletion efficiency was determined by flow cytometric analysis of ankle and spleen tissue. To block MHC-II, mice were intraperitoneally inoculated with 200 μg of either isotype control antibody (LTF-2; Bioxcell) or anti-MHC-II antibody (M5/114; Bioxcell) every 3 days starting at 14 dpi. Mice were killed at 28 dpi. To inhibit CHIKV RNA replication in vivo, at 28 dpi mice were administered vehicle only (10% 1-methyl-2-pyrrolidone, 30% saline and 60% polyethylene glycol) or 60 mg kg^−1^ of SRI-42718 in vehicle by oral gavage every 12 hours for 7 days. At 35 dpi, tissues were collected and analysed as outlined next.

### Preparation of joint tissue single-cell suspensions

Single-cell suspensions were generated from joint-associated tissues by mechanical and enzymatic digestion as previously described^46,47^. At the indicated time points, the ipsilateral ankle and foot tissue were dissected. Tissues were horizontally agitated with 6.0 mm glass beads in RPMI 1640 (Gibco) medium supplemented with 10% FBS, 15 mM HEPES, 2.5 mg ml^−1^ collagenase A (Roche) and 1.7 mg ml^−1^ DNase I (Sigma) for 2 hours at 37 °C. After incubation, digested tissues were filtered through a 70-μm cell strainer, cells were washed in wash buffer (1× Hanks Balanced Salt Solution and 15 mM HEPES) and total viable cells were determined by trypan blue exclusion.

### Single-cell mRNA sequencing

#### Single-cell library preparation using the 10x Genomics platform

We targeted recovery of 10,000 cells for scRNA-seq in each sample. Final cell suspensions were emulsified, lysed and barcoded using the Next GEM Chip G Kit (1000127) and a 10x Genomics chromium controller housed in a BSL-3 laboratory. Single-cell gene expression libraries were generated using the Next GEM Single Cell 3′ GEM, Library and Gel Bead Kit v.3.1 (1000128) and Single Index Kit T Set A (1000213) according to the manufacturer’s instructions (10x Genomics). Sequences were generated with the Illumina NovaSEQ 6000 instrument using S4 flow cells and 300 cycle SBS reagents. We targeted 50,000 reads per cell, with the following sequencing parameters: read 1, 151 cycles; i7 index, 8 cycles; i5 index, 0 cycles; read 2, 151 cycles.

#### CHIKV-capture library preparation

The scRNA-seq libraries were enriched for molecules aligning to the CHIKV genome according to our previously published methods^90^. Briefly, the CHIKV genome was PCR amplified in 3 fragments (primer sequences: CHIKV-F1, 5′-TGAGACACACGTAGCCTACCA-3′; CHIKV-F2, 5′-AAGTCCAAGGGAATACAGATCTTC-3′; CHIKV-F3, 5′-ACCGCAGCACGGTAGAGA-3′; CHIKV-R1, 5′-CGAATAACATTACCTTGGAGCA-3′; CHIKV-R2, 5′-TTTTTCCCGGCCTATCACAG-3′; and CHIKV-R3, 5′-AAAAACAAAATAACATCTCCTACGTC-3′) and labelled with biotin-dUTP using the same primers before sonicating to generate ~150 bp fragments for hybridization. Denatured and diluted biotin-dUTP-labelled CHIKV genome fragments were hybridized to the concentrated scRNA-seq libraries separately. Streptavidin capture beads were mixed with the hybridized libraries and washed to remove unbound DNA. Libraries were amplified directly from the cleaned-up beads and sequenced as described earlier. FASTQ files for each replicate (three mock and three CHIKV infected) were processed using the cellranger count pipeline (v.6.0.1).

#### scRNA-seq gene expression analysis

FASTQ files for each replicate (three mock- and three CHIKV-infected) were processed using the cellranger count pipeline (v.6.0.1). Reads were aligned to a version of the mm10 reference genome that also included the CHIKV AF15561 (EF452493.1) genome. The CHIKV genome included annotations for the subgenomic RNA (position 7,567–12,036) and 5′ (position 1–7,566) regions. Initial filtering of gene expression data was performed separately for each biological replicate using the Seurat R package (v.4.3.1). CHIKV counts were excluded from the gene expression matrices so that they would not influence downstream processing (dimensionality reduction and clustering) of the mouse expression data. Cells were filtered based on the number of detected mouse genes (>250 and <5,500) and the per cent of mitochondrial counts (<30%). Genes were filtered to only include those detected in more than five cells. Potential cell doublets were removed using the DoubletFinder (v.2.0.3) R package. Mouse gene expression counts were normalized by the total mouse counts for the cell, multiplied by a scale factor (10,000) and log transformed (NormalizeData). The gene expression data for each biological replicate were combined into a single Seurat object. The normalized mouse counts were scaled and centred (ScaleData) using the top variable features identified with the M3Drop (v.1.28.0) R package. The scaled data were used for principal component analysis (RunPCA), and the first 50 principal components were used to identify clusters (FindNeighbors and FindClusters) and calculate uniform manifold approximation and projection (UMAP; RunUMAP). Counts from the CHIKV-capture libraries were then added to the object for all cells passing our filtering cut-offs. CHIKV^+^ cells were classified as any cell with at least one CHIKV RNA count in either the CHIKV-capture or bulk scRNA-seq libraries.

We generated an initial set of broad cell type annotations using the R package clustifyr^91^ (v.1.14.0) and reference data from Immgen^92^. These annotations were checked for accuracy and further refined using known cell type markers. To annotate macrophage subsets, macrophages were reclustered and samples were integrated using the R package Harmony (v.1.2.0)^93^. Macrophage clusters were annotated using published marker genes^54^. To annotate T cell subsets, T cells were reclustered, samples were integrated using the Harmony package and clusters were annotated using known marker genes.

DEGs were identified for each cell subset for mock- versus CHIKV-infected samples using the Seurat R package. *P* values were adjusted for multiple testing using the Benjamini–Hochberg method. DEGs were filtered to only include those with an absolute log_2_-transformed fold change >0.25 for all 3 replicates and a maximum adjusted *P* < 0.05. Mitochondrial genes were excluded. DEGs were identified for all macrophages and T cell subsets from mock- versus CHIKV-infected samples using the Seurat package. Due to the low number of T cells, all replicates were grouped together. GSEA was performed using the clusterProfiler (v.4.10.0) R package^94^. *P* values were calculated using an adaptive multilevel split Monte Carlo method. Genes were included that were expressed in at least 10% of cells for the mock- or CHIKV-infected samples. These genes were ranked based on the log-transformed fold change in average expression and compared with disease ontology terms from the DOSE R package (v.3.28.2). GSEA terms were filtered to remove broad terms that include >400 genes. *P* values were adjusted for multiple testing using the Benjamini–Hochberg method and terms were filtered for those with an adjusted *P* value < 0.05.

Changes in the abundance of different macrophage subsets were assessed using the propeller method available from the speckle R package (v.1.2.0)^95^. False-discovery-rate-adjusted *P* values were calculated using moderated *t*-tests performed on logit-transformed, variance stabilized cell type proportions.

### RNA isolation and RT-qPCR quantification of RNA from joint-associated tissue

To quantify gene expression and viral RNA in tissues, ankle tissue was dissected from mice and homogenized in Trizol using a FastPrep-24 Classic homogenizer (MP Biomedical). Total RNA was isolated using a PureLink RNA Mini Kit (Life Technologies) and cDNA was generated using random hexamers and SuperScript IV reverse transcriptase (Life Technologies). All primers were obtained from Integrated DNA Technologies. CHIKV RNA was quantified by qPCR with CHIKV-specific forward (5′-TTTGCGTGCCACTCTGG-3′) and reverse (5′-CGGGTCACCACAAAGTACAA-3′) primers, with an internal TaqMan probe (5′-ACTTGCTTGATCGCCTTGGTGAGA-3′), all within the nsP2 gene region, as previously described^46^. MAYV RNA was quantified using MAYV-specific forward (5′-AAGCTCTTCCTCTGCATTGC-3′) and reverse (5′-TGCTGGAAACGCTCTCTGTA-3′) primers, with an internal TaqMan probe (5′- GCCGAGAGCCCGTTTTTAAAATCA-3′) within the nsP1 gene region. RRV RNA was amplified through the generation of cDNA with a sequence-tagged (in lowercase letters) RRV-specific RT primer (5′-ggcagtatcgtgaattcgatgcAACACTCCCGTCGACAACAGA-3′) and quantified by qPCR with an RRV-specific forward primer (5′-CCGTGGCGGGTATTATCAAT-3′) and a tag sequence-specific reverse primer (5′-GGCAGTATCGTGAATTCGATGC-3′), with an internal TaqMan probe (5′-ATTAAGAGTGTAGCCATCC-3′) within the nsP3 gene region. The relative fold of host gene expression between mock- and CHIKV-infected samples was determined using the comparative Ct method^96^ and commercially available RT-qPCR TaqMan Gene Expression Assays (Thermo Fisher Scientific): IL-1β (Mm00434229_m1), TNF (Mm00443258_m1), H2-Aa (Mm00439211_m1), IFNγ (Mm01168134_m1) and NLRP3 (Mm00840904_m1). GAPDH (Mm99999915_g1) was used as a control to normalize for input amounts of cDNA.

### RNA isolation and RT-qPCR from fixed-sorted or non-fixed cells

To sort cells from joint-associated tissue, single-cell suspensions were stained for flow cytometry as described above and fixed in 2% PFA in PBS for 10 minutes at room temperature. Samples were sorted using a MoFlo XDP100 (Beckman Coulter) flow cytometer. RNA was extracted from fixed-sorted cells with the RNeasy FFPE Kit (Qiagen) and cDNA was generated using random hexamers and SuperScript IV reverse transcriptase (18091200; Life Technologies). CHIKV, MAYV or RRV nucleic acid and host gene copies were quantified using the previously described methods. To isolate CD11b^+^ cells from all other cells in joint-associated tissue, single-cell suspensions were generated as previously described and mononuclear cells were isolated by density gradient centrifugation using Lympholyte-M Cell Separation Media (CL5031; Cedarlane). Purified cells were labelled with CD11b magnetic beads (130-049-601; Miltenyi Biotec) and flowed through a MACS column to capture labelled cells. Eluted labelled cells were either lysed in Trizol for RT-qPCR analysis of cellular RNA or stained for analysis by flow cytometry. All flow-through fractions were combined with the pelleted cells from the Lympholyte-M purification to generate the ‘all other cells’ fractions.

### Deep sequencing of CHIKV RNA

#### Sample sequencing

Viral whole genome sequencing (WGS) of CHIKV inoculum from viral stocks was performed using a metagenomic next-generation sequencing protocol as described previously^97^. Briefly, extracted RNA was treated with a TURBO DNA-free Kit (AM1907; Thermo Fisher) and reverse transcribed using random hexamers (N8080127; Thermo Fisher) and SuperScript IV (18090010; Thermo Fisher) according to the manufacturer’s protocol. Double-stranded cDNA synthesis was performed using Sequenase v.2.0 (70775Z1000UN; Thermo Fisher) and the resulting cDNA was purified using 1.8× AMPure XP magnetic beads (A63882; Beckman Coulter). Metagenomic viral WGS libraries were created from purified double-stranded cDNA with tagmentation reagents from the Illumina DNA Prep with Enrichment Kit (20060059; Illumina), followed by 18 cycles of dual-indexed PCR. Amplified libraries were cleaned with 0.8× AMPure XP magnetic beads. Viral isolate libraries were not subjected to enrichment–hybridization capture due to high viral content. Viral WGS was also performed on three joint-associated tissues and three sorted joint tissue macrophage specimens. These samples were processed using QIAseq xHYB Microbial Hyb&Lib Kit A (334525; Qiagen) and a custom Qiagen hybridization panel synthesized based on CHIKV strain AF15561 (EF452493.1) following the manufacturer’s protocol. In short, extracted RNA was depleted of mouse ribosomal RNA, converted to double-stranded cDNA, enzymatically fragmented, end repaired, indexed, purified using 0.9× and 1.1× bead clean-ups and amplified with 14 cycles of PCR followed by a final bead purification. Precapture libraries were then pooled based on RNA copies per microgram of RNA (one to three samples per pool) and hybridized overnight with a custom biotinylated probe panel. Probe-bound targets were captured using streptavidin-coated magnetic beads and washed to remove non-specific fragments. Enriched libraries were amplified with 20 cycles of PCR and purified using a 1.1× bead clean-up. Final library concentrations were quantified with the Qubit 4 Fluorometer and dsDNA HS Assay Kit. All libraries were sequenced on Illumina NextSeq 2000 instruments using a 2 × 150 bp read format.

#### Data analysis

Raw sequencing reads were trimmed and quality filtered using fastp (v.0.23.4) with the following settings: --cut_mean_quality 20 --cut_front --cut_tail --length_required 20 --low_complexity_filter --trim_poly_g --trim_poly_x. Quality-controlled reads from the viral inoculum stocks were randomly downsampled to 1 million paired-end reads using seqtk (v.1.4). Consensus genomes for the inoculum stocks were generated by majority rule in Geneious Prime 2025.0, using the CHIKV strain AF15561 (EF452493.1) as the reference. Variant calling was performed with the RAVA pipeline (default settings), using the matched inoculum consensus as a reference (https://github.com/greninger-lab/RAVA_Pipeline/tree/2024-12-09_CU_KZ_CHIKV_publication).

### Flow cytometry

Single-cell suspensions were blocked with anti-FcγRIII/II (2.4G2; BD Pharmingen) for 10 minutes, stained with LIVE/DEAD Fixable Violet Dead Cell Stain (Invitrogen) according to product instructions and then stained with the indicated antibodies for 45 minutes on ice. Cells were washed 2× with FACS buffer (1% FBS, 2 µM EDTA, 20 mM HEPES in 1× PBS) and fixed by addition of PFA to a final concentration of 1% for 10 minutes at room temperature. For intracellular stains, fixed cells were incubated with the indicated antibodies in 0.1% saponin in FACS buffer for 0.5–2 hours at room temperature, washed 3× with 0.1% saponin in FACS buffer and resuspended in FACS buffer. Samples were acquired using a Cytek Aurora cytometer (>50,000 events) and Aurora software. Downstream analysis was performed using FlowJo software (Tree Star). All antibodies were purchased from BioLegend unless otherwise indicated: CD11b (M1-70), CD11c (N418), TCRβ (H57-597), CD3ε (145-2C11), CD4 (GK1.5), CD8 (53-6.7), MHC-II (IA/IE; M5/114.15.2; eBioscience or BD Biosciences), F4/80 (BM8), CD45 (30-F11; BioLegend or BD Biosciences), NK1.1 (PK136), Ly6G (1A8), Ly6C (HK1.4), B220 (RA3-6B2), IFNγ (XMG12) and CD90.2 (30-H12; Invitrogen).

### Ex vivo T cell stimulation

Single-cell suspensions of joint-associated tissue collected at 28 dpi were stimulated ex vivo with the MHC class I OVA peptide, OVA-257 (1 μg) or with an E1 peptide pool^66^ (1 μg) in the presence of 3 μg ml^−1^ brefeldin A for 5 hours and then processed for surface staining as previously described. Following surface staining and PFA fixation, cells were washed 2× in saponin buffer (1 mg ml^−1^ saponin in FACS buffer). For intracellular staining, cells were resuspended in a cocktail of intracellular antibodies in saponin buffer for 2 hours at room temperature. After incubation, cells were washed twice with saponin buffer and acquired on the Cytek Aurora cytometer.

### Spatial transcriptomics

#### Tissue processing

Paraffin-embedded mouse ankle blocks were sectioned on a microtome under RNAse-free conditions to prepare slides for Xenium analysis. Sections (5 µm) were placed onto Xenium slides (PN-3000941; 10x Genomics), with 3 animal’s ankles included per slide, and allowed to airdry overnight in a slide desiccator. Slides were then baked for 2 hours at 60 °C, followed by deparaffinization and decrosslinking. Panels of oligonucleotide probes including the 379 gene Xenium Mouse Tissue Atlassing Panel (PN-2000949; 10x Genomics) and a custom panel of 98 genes (design identifier QX42XM) were hybridized to RNA targets in situ. Our custom gene panel included targets for the CHIKV genome, with separate probes for 3′ (position 7,567–12,036) and 5′ (position 1–7,566) regions, and for negative-strand CHIKV RNA. Rolling circle amplification was used to boost the fluorescence signal following the steps outlined in the Xenium In Situ Gene Expression User Guide (CG000749 Rev B). Slides were loaded onto the Xenium Analyzer (PN-1000569; 10x Genomics) for imaging and analysis. Regions of interest were manually selected, one region per sample, before capture. Cell segmentation staining reagents (PN-1000661) were applied to the tissue as described in the user guide (CG000749 Rev B) to aid in accurate assignment of cell boundaries during analysis.

#### Data analysis

Data for each tissue section were processed as a separate ‘field of view’ (FOV) using the Seurat R package (v.4.4.2). For each slide, a separate Seurat object was generated for each FOV. CHIKV counts were added to the objects as a separate assay. Gene expression counts for each cell were normalized (SCTransform) and principal component analysis and UMAP were performed (RunPCA and RunUMAP), followed by clustering (FindNeighbors and FindClusters). To annotate cell types, we first generated a cell type reference using our scRNA-seq data for the mock-infected replicate with the greatest number of cells. We then transferred cell type labels to each Xenium FOV object using Seurat (FindTransferAnchors and TransferData). Cells with a cell type prediction confidence ≤0.5 were labelled as unassigned. CHIKV^+^ cells were identified as any cell with at least one CHIKV count. To assess cell–cell proximity enrichment for CHIKV^+/−^ cells, we used the Giotto R package (v.4.2.1)^98^. For each FOV, we created a separate Giotto object (createGiottoXeniumObject) containing cell type annotations for CHIKV^+^ and CHIKV^−^ cells. We then created a spatial Delaunay network (createSpatialDelaunayNetwork) and computed cell–cell interaction enrichment (cellProximityEnrichment). Cell types were only included if they had ≥20 CHIKV^+^ cells for all 3 biological replicates. Interaction results were filtered to only include interactions with *P* < 0.05.

### Quantification and statistical analysis

Data collection and analysis were not performed blind to the conditions of the experiments. No data points were excluded from the analyses. Flow cytometry and RT-qPCR data were analysed using GraphPad Prism v.10.5.0 software. Column heights indicate mean values; error bars indicate the s.e.m. Box plots were drawn as follows: centre line indicate the median; box limits, upper and lower quartiles; whiskers, 1.5× interquartile range; and points, outliers. For statistical analysis, data were evaluated using either one-way analysis of variance (ANOVA) with multiple comparisons test or Student’s *t*-test. *P* < 0.05 was considered significant. All differences not indicated as significant had *P* > 0.05. The number of replicates per experiment and the *P* values are indicated in the figure legends. Data distribution was assumed to be normal but this was not formally tested.

### Ethics statement

This study was conducted in accordance with the recommendations in the Guide for the Care and Use of Laboratory Animals and the American Veterinary Medical Association Guidelines for the Euthanasia of Animals. All animal experiments were performed with the approval of the Institutional Animal Care and Use Committee of the University of Colorado Anschutz (assurance number A3269-01) under protocols 00026 and 00215. Experimental animals were humanely euthanized at defined endpoints by exposure to isoflurane vapours followed by bilateral thoracotomy.

### Reporting summary

Further information on research design is available in the Nature Portfolio Reporting Summary linked to this article.

## Supplementary information

Supplementary Information Supplementary Figs. 1–6.

Reporting Summary

Supplementary Data 1 Source data.

Supplementary Data 2 Source data.

Supplementary Data 3 Source data.

## Source data

Source Data Fig. 1 Statistical source data.

Source Data Fig. 2 Statistical source data.

Source Data Fig. 3 Statistical source data.

Source Data Fig. 4 Statistical source data.

Source Data Fig. 6 Statistical source data.

Source Data Extended Data Fig. 1 Statistical source data.

Source Data Extended Data Fig. 2 Statistical source data.

Source Data Extended Data Fig. 3 Statistical source data.

Source Data Extended Data Fig. 4 Statistical source data.

Source Data Extended Data Fig. 5 Statistical source data.

Source Data Extended Data Fig. 6 Statistical source data.

## Data Availability

The authors declare that all data supporting the findings of this study are available within the paper, its extended data or source data files. The scRNA-seq and spatial transcriptomics data have been deposited in the NCBI GEO database (GSE300299, GSE300303). Raw CHIKV RNA-sequencing data are available at NCBI BioProject PRJNA1247591 (https://www.ncbi.nlm.nih.gov/bioproject/?term=PRJNA1247591). Source data are provided with this paper.

## Code availability

Analysis pipelines are available via Zenodo at 10.5281/zenodo.18499812 (ref. ^99^) and 10.5281/zenodo.18499817 (ref. ^100^). An analysis pipeline for the WGS is available via Github at https://github.com/greninger-lab/RAVA_Pipeline/tree/2024-12-09_CU_KZ_CHIKV_publication.
