# Supplementary material for: Chikungunya virus persists in joint-associated macrophages and promotes chronic disease in mice
Source: Nat Microbiol. 2026 Apr 1;11(5):1302–17. doi: 10.1038/s41564-026-02303-9 (PMC13171603; doi:10.1038/s41564-026-02303-9)
Supplement: Supplementary file 1 — Supplementary Figs. 1–6. [file 41564_2026_2303_MOESM1_ESM.pdf]

nature microbiology

Supplementary information https://doi.org/10.1038/s41564-026-02303-9

Chikungunya virus persists in joint-associated macrophages and promotes chronic disease in mice

In the format provided by the authors and unedited

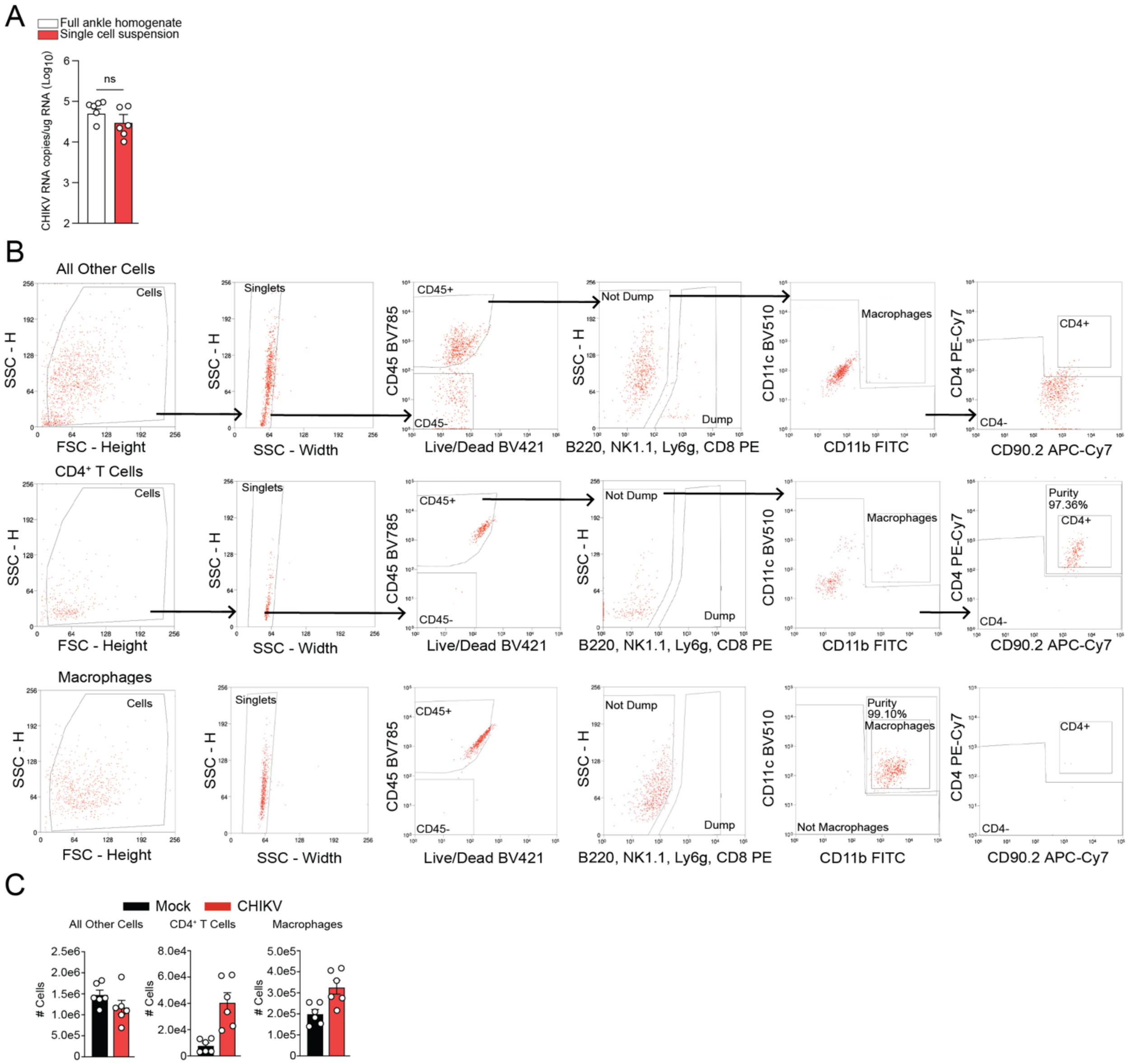

Supplementary Figure 1. Cell sorting strategy for macrophages, CD4⁺ T cells, and all other cells from joint-associated tissue during chronic CHIKV disease. (**A**) WT C57BL/6 mice were inoculated with PBS (mock; n=6) or 10^3 PFU CHIKV (n=6) in the left rear footpad. At 28 dpi, CHIKV RNA copies in whole ankle homogenates or homogenates of single cell suspensions generated from enzymatically digested ankle tissue were quantified by RT-qPCR. (**B**) Representative flow cytometry plots demonstrating cell gates used to sort designated cell populations as well as the purity of each sorted cell population. (**C**) The total number of cells sorted for each of the cell populations from mock- (n=6) and CHIKV-infected (n=6) mice. Data are from 2 independent experiments (A, C) while flow plots are representative of 2 independent experiments comprised of 3 replicates each (B). Data are presented as mean values +/- SEM. *P* values were determined by two-sided unpaired Student's t test (A).

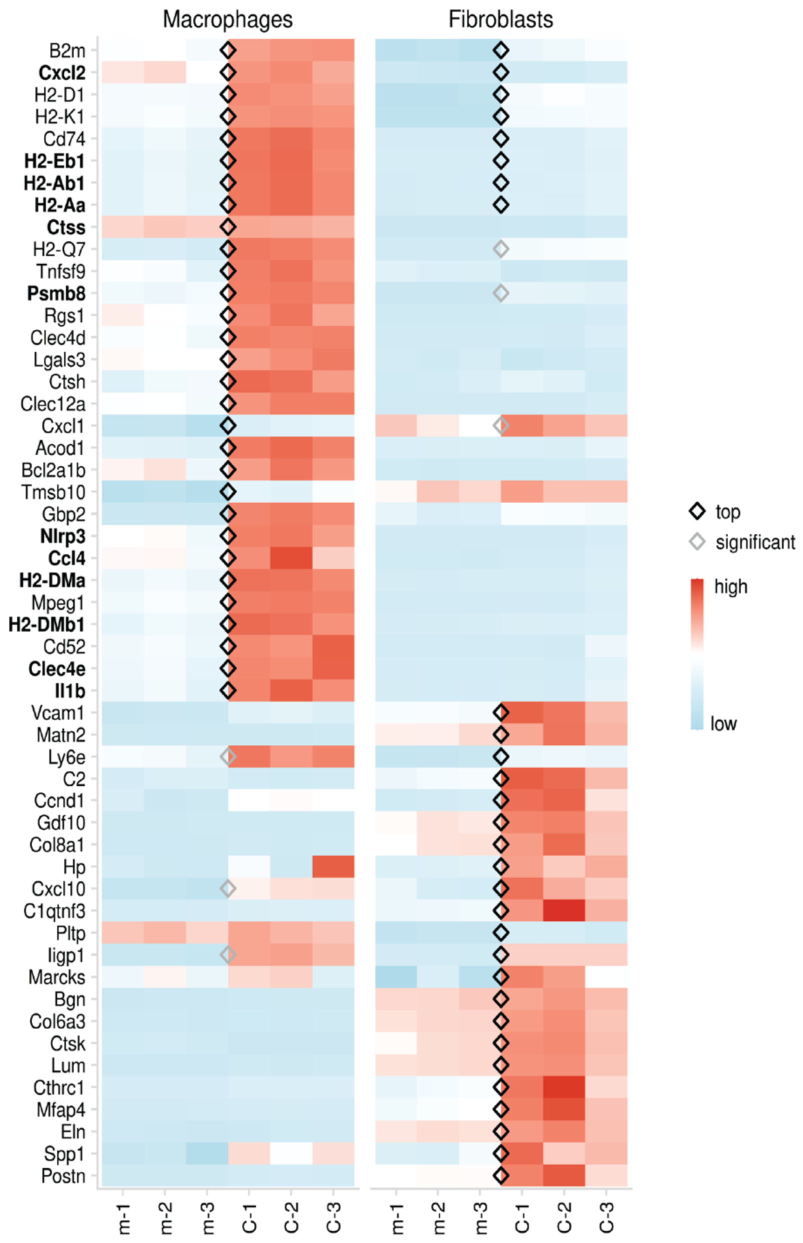

Supplementary Figure 2. Joint tissue fibroblasts display a pro-inflammatory gene expression profile. WT C57BL/6 mice were inoculated with PBS (mock; n=3) or 10^3 PFU CHIKV (n=3) in the left rear footpad. At 28 dpi, ankle joint-associated single cells were analyzed by scRNA-seq. Heatmap shows genes upregulated in macrophages and fibroblasts from CHIKV-infected mice at 28 dpi. Genes with a black diamond are significantly upregulated and are within the top 30 upregulated genes for the cell type. Genes with a grey diamond are significantly upregulated for the cell type but are not within the top 30 upregulated genes. Genes were ordered first based on whether they were shared between both cell types, then based on significance for the cell type in which the gene is upregulated. Genes of interest are shown in **bold**.

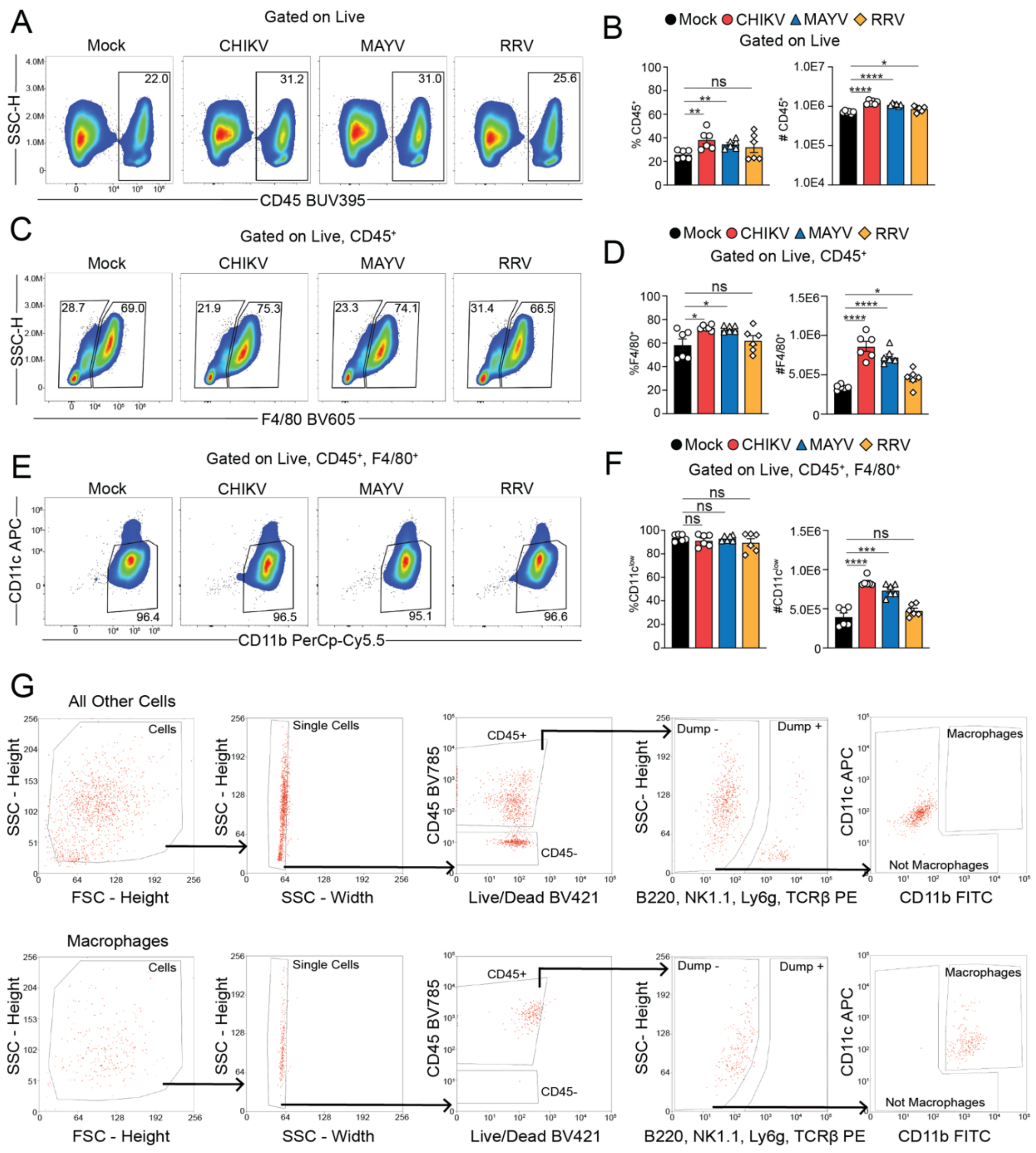

Supplementary Figure 3. Macrophages are cellular sites of viral RNA persistence during CHIKV, MAYV, and RRV infection. (**A-F**) WT C57BL/6 mice were inoculated with PBS (mock; n=6) or 10^3 PFU CHIKV (n=6), MAYV (n=6), or RRV (n=6) in the left rear footpad. At 28 dpi, ankle joint-associated single cells were assessed by flow cytometry. (**A**) Representative flow cytometry plots of $CD45^+$ cells among live, singlet cells. (**B**) Frequency and number of $CD45^+$ cells. (**C**) Representative flow cytometry plots of $F4/80^+$ cells among live, singlet, $CD45^+$ cells. (**D**) Frequency and number of $F4/80^+$ cells. (**E**) Representative flow cytometry plots of $CD11b^+$ and $CD11c^+$ cells among live, singlet, $CD45^+$, $F4/80^+$ cells. (**F**) Frequency and number of $CD11c^{low}$ cells. (**G**) FACS gating strategy for sorting all other cells and macrophages from joint-associated tissue. Data are representative of 2 independent experiments. Data are presented as mean values +/- SEM. *P* values were determined by two-sided unpaired Student's t test (B, D, F). *, $P < 0.05$; **, $P < 0.01$; ***, $P < 0.001$; ****, $P < 0.0001$.

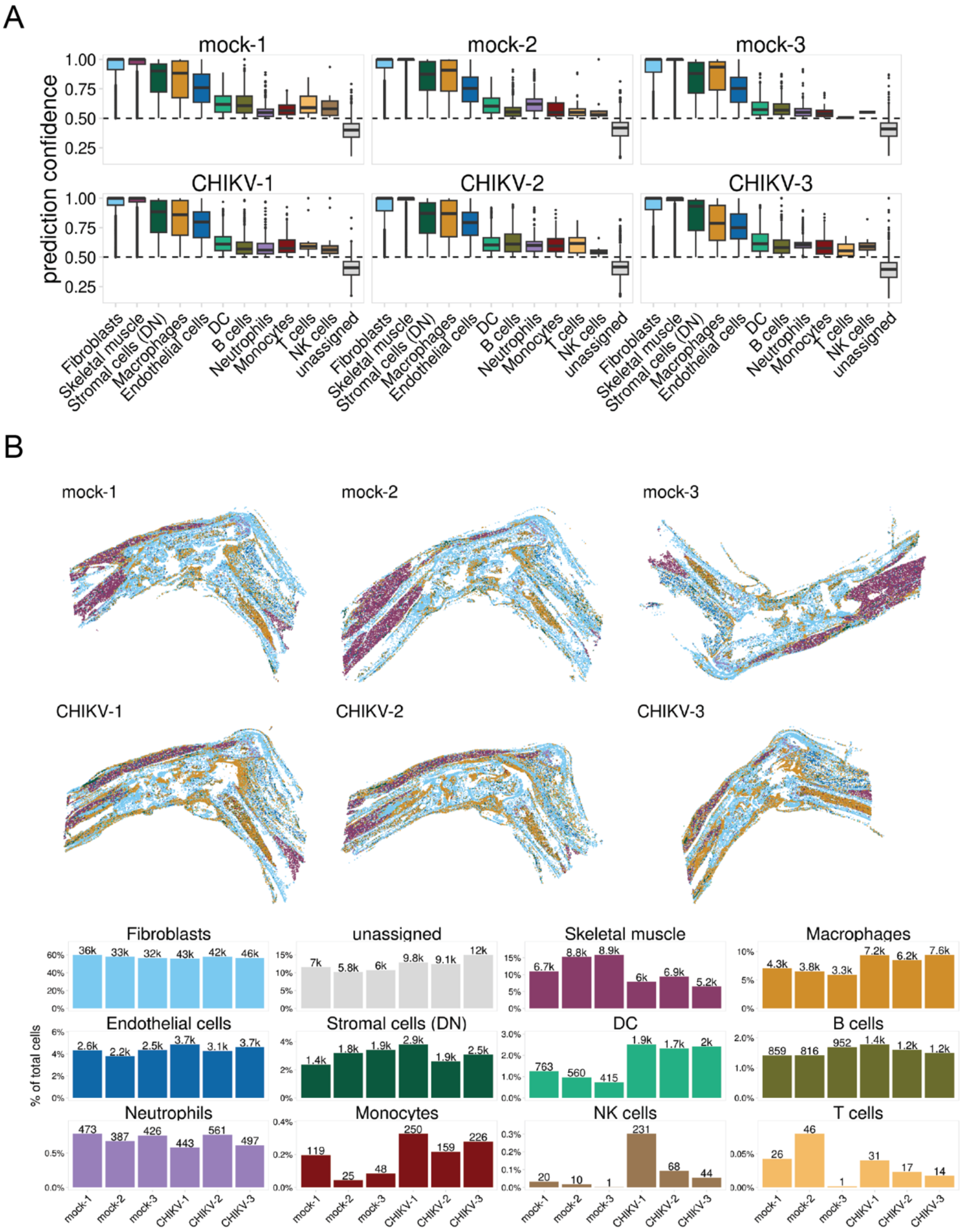

Supplementary Figure 4. Spatial transcriptomics: cell type annotation. (**A-B**) WT C57BL/6 mice were inoculated with PBS (mock; n=3) or 10^3 PFU CHIKV (n=3) in the left rear footpad. At 28 dpi, joint-associated tissue was analyzed by spatial transcriptomics. (**A**) Per cell predication confidence for cell type annotations. (**B**) Annotated cell types shown for each tissue section. Bar graphs display the percent of each cell type among total cells and the total number of each cell type detected in each independent tissue section.

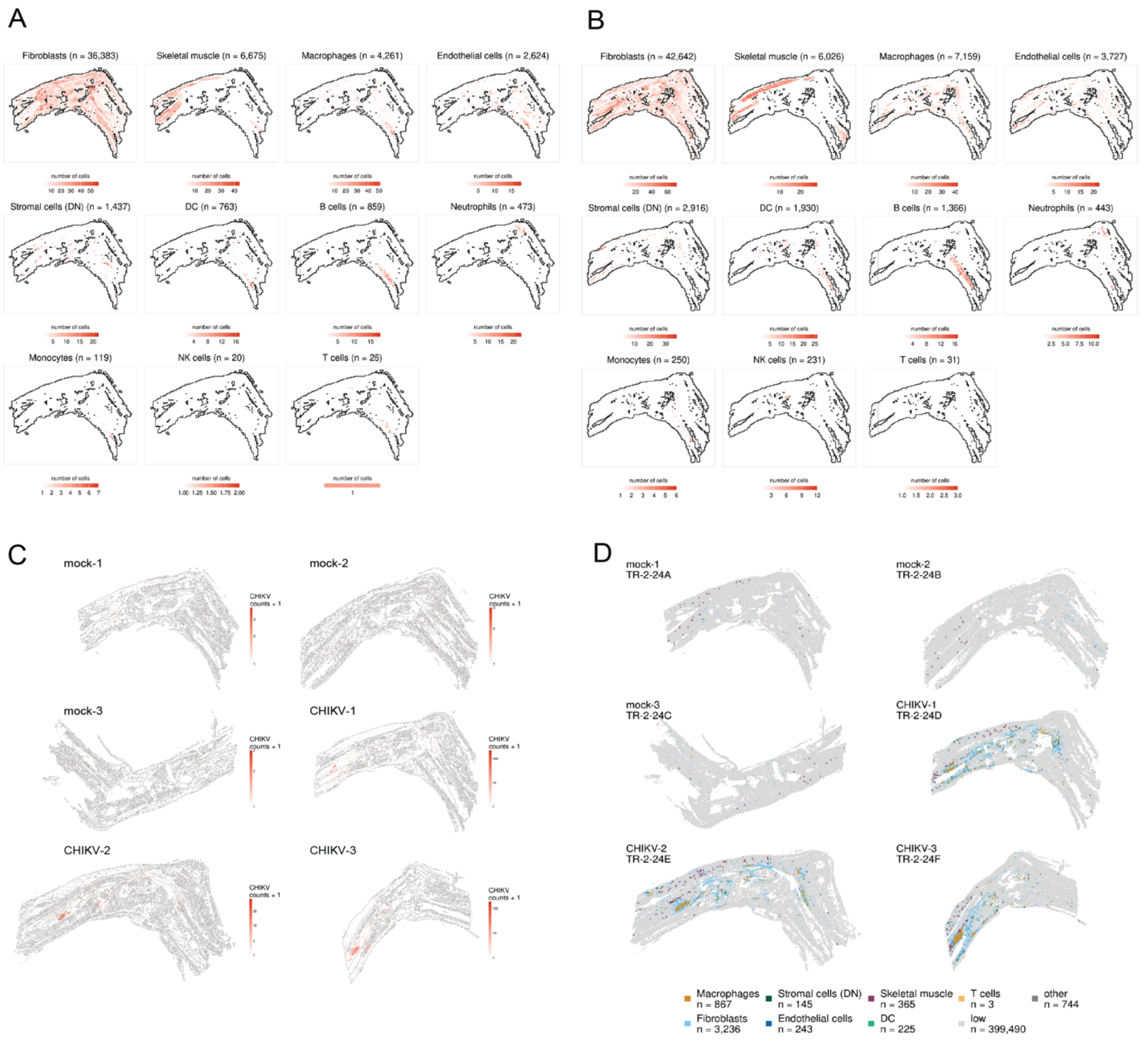

Supplementary Figure 5. Spatial transcriptomics: spatial distribution of cell types and CHIKV RNA$^+$ cells. (**A-D**) WT C57BL/6 mice were inoculated with PBS (mock; n=3) or 10^3 PFU CHIKV (n=3) in the left rear footpad. At 28 dpi, joint-associated tissue was analyzed by spatial transcriptomics. (**A**) Representative heatmaps showing the spatial distribution of annotated cell types in tissue from mock-infected mice. (**B**) Representative heatmaps showing the spatial distribution of annotated cell types in tissue from CHIKV-infected mice. (**C**) CHIKV RNA counts in cells of mock- and CHIKV-infected joint-associated tissue are shown for each tissue section. (**D**) CHIKV RNA$^+$ cells (>0 total CHIKV counts) are shown for each tissue section.

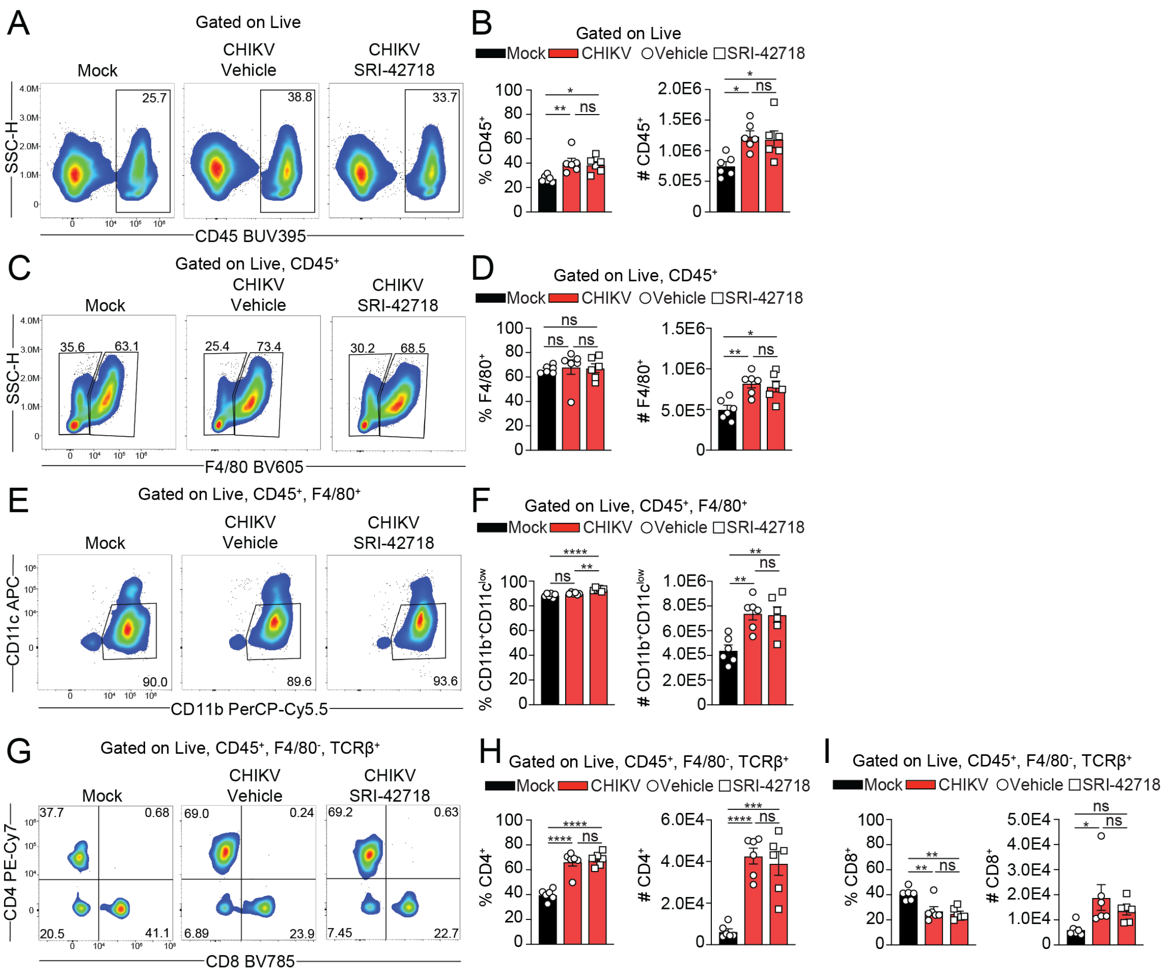

Supplementary Figure 6. Evaluation of joint cell populations following treatment with a small molecule antiviral during chronic CHIKV disease. (**A-I**) WT C57BL/6 mice were inoculated with PBS (mock; n=8) or 10^3 PFU CHIKV (n=8) in the left rear footpad. At 28 dpi, mice were administered 60 mg/kg of a small molecule antiviral, SRI-42718, by oral gavage every 12 h for 7 days. Infected control mice were treated with vehicle alone. At 35 dpi, ankle joint-associated tissue was isolated from a subset of the mice for analysis by flow cytometry. (**A**) Representative flow cytometry plots of $CD45^+$ cells among live, singlet cells. (**B**) Frequency and number of $CD45^+$ cells. (**C**) Representative flow cytometry plots of $F4/80^+$ cells among live, singlet, $CD45^+$ cells. (**D**) Frequency and number of $F4/80^+$ cells. (**E**) Representative flow cytometry plots of $CD11b^+$ and $CD11c^+$ cells among live, singlet, $CD45^+$, $F4/80^+$ cells. (**F**) Frequency and number of $CD11b^+CD11c^{low}$ cells among live, singlet, $CD45^+$, $F4/80^+$ cells. (**G**) Representative flow cytometry plots of $CD4^+$ and $CD8^+$ T cells among live, singlet, $CD45^+$, $F4/80^-$, $TCR\beta^+$ cells. (**H-I**) Frequency and number of $CD4^+$ or $CD8^+$ T cells. Data are representative of 2 independent experiments. Data are presented as mean values +/- SEM. *P* values were determined by two-sided one-way ANOVA with Tukey's multiple comparisons test. *, $P < 0.05$; **, $P < 0.01$; ***, $P < 0.001$; ****, $P < 0.0001$.
