# Supplementary material for: Chikungunya virus persists in joint-associated macrophages and promotes chronic disease in mice
Source: Nat Microbiol. 2026 Apr 1;11(5):1302–17. doi: 10.1038/s41564-026-02303-9 (PMC13171603; doi:10.1038/s41564-026-02303-9)
Supplement: Supplementary file 2 — Reporting Summary [file 41564_2026_2303_MOESM2_ESM.pdf]

nature portfolio

Corresponding author(s): Thomas E.Morrison

Last updated by author(s): Feb 9, 2026

# Reporting Summary

Nature Portfolio wishes to improve the reproducibility of the work that we publish. This form provides structure for consistency and transparency in reporting. For further information on Nature Portfolio policies, see our Editorial Policies and the Editorial Policy Checklist.

## Statistics

For all statistical analyses, confirm that the following items are present in the figure legend, table legend, main text, or Methods section.

| n/a | Confirmed | |
|---|---|---|
| ☐ | ☒ | The exact sample size (*n*) for each experimental group/condition, given as a discrete number and unit of measurement |
| ☐ | ☒ | A statement on whether measurements were taken from distinct samples or whether the same sample was measured repeatedly |
| ☐ | ☒ | The statistical test(s) used AND whether they are one- or two-sided<br>*Only common tests should be described solely by name; describe more complex techniques in the Methods section.* |
| ☒ | ☐ | A description of all covariates tested |
| ☐ | ☒ | A description of any assumptions or corrections, such as tests of normality and adjustment for multiple comparisons |
| ☐ | ☒ | A full description of the statistical parameters including central tendency (e.g. means) or other basic estimates (e.g. regression coefficient) AND variation (e.g. standard deviation) or associated estimates of uncertainty (e.g. confidence intervals) |
| ☐ | ☒ | For null hypothesis testing, the test statistic (e.g. *F*, *t*, *r*) with confidence intervals, effect sizes, degrees of freedom and *P* value noted<br>*Give P values as exact values whenever suitable.* |
| ☒ | ☐ | For Bayesian analysis, information on the choice of priors and Markov chain Monte Carlo settings |
| ☒ | ☐ | For hierarchical and complex designs, identification of the appropriate level for tests and full reporting of outcomes |
| ☒ | ☐ | Estimates of effect sizes (e.g. Cohen's *d*, Pearson's *r*), indicating how they were calculated |

*Our web collection on statistics for biologists contains articles on many of the points above.*

## Software and code

Policy information about availability of computer code

| | |
|---|---|
| Data collection | Flow cytometry data collection was performed using SpectroFlo software on the Aurora (Cytek). Flow sorting data collection was performed using Summit sofware on the XDP Flex (Beckman Coulter). |
| Data analysis | GraphPad Prism v10.5 and v10.6.1 were used to perform statistical analysis. FlowJo software v10.10 was used to analyze flow cytometry data. FASTQ files for scRNA-seq data were processed using the Cell Ranger count pipeline (v6.0.1). Downstream analysis of scRNA-seq data was performed in R 4.3.1 using the Seurat R package (v4.4.0). Samples were integrated using the Harmony R package (v1.1.0). Cell types were annotated using the clustifyr R package (v1.14.0). Differentially expressed genes were identified using Seurat. Plots were generated using the ggplot2 R package (3.5.1). 10X Xenium data was processed in R 4.4.2 using the Seurat R package (v5.2.0). Cell-cell proximity enrichment analysis was performed using the Giotto R package (v4.2.1). Analysis pipelines are available at https://doi.org/10.5281/zenodo.18499812 and https://doi.org/10.5281/zenodo.18499817.. An analysis pipeline for the WGS is available at https://github.com/greninger-lab/RAVA_Pipeline/tree/2024-12-09_CU_KZ_CHIKV_publication. |

For manuscripts utilizing custom algorithms or software that are central to the research but not yet described in published literature, software must be made available to editors and reviewers. We strongly encourage code deposition in a community repository (e.g. GitHub). See the Nature Portfolio guidelines for submitting code & software for further information.

## Data

Policy information about availability of data

All manuscripts must include a data availability statement. This statement should provide the following information, where applicable:

- Accession codes, unique identifiers, or web links for publicly available datasets
- A description of any restrictions on data availability
- For clinical datasets or third party data, please ensure that the statement adheres to our policy

The authors declare that all data supporting the findings of this study are available within the paper, its Extended Data, or Source Data files. The scRNA-seq and spatial transcriptomics data have been deposited in the NCBI GEO database (GSE300299, GSE300303). Raw CHIKV RNA sequencing data is available at NCBI BioProject PRJNA1247591 (https://www.ncbi.nlm.nih.gov/bioproject/?term=PRJNA1247591).

## Research involving human participants, their data, or biological material

Policy information about studies with human participants or human data. See also policy information about sex, gender (identity/presentation), and sexual orientation and race, ethnicity and racism.

| | |
|---|---|
| Reporting on sex and gender | N/A |
| Reporting on race, ethnicity, or other socially relevant groupings | N/A |
| Population characteristics | N/A |
| Recruitment | N/A |
| Ethics oversight | N/A |

Note that full information on the approval of the study protocol must also be provided in the manuscript.

# Field-specific reporting

Please select the one below that is the best fit for your research. If you are not sure, read the appropriate sections before making your selection.

☒ Life sciences ☐ Behavioural & social sciences ☐ Ecological, evolutionary & environmental sciences

For a reference copy of the document with all sections, see nature.com/documents/nr-reporting-summary-flat.pdf

# Life sciences study design

All studies must disclose on these points even when the disclosure is negative.

| | |
|---|---|
| Sample size | No sample sizes were chosen a priori. All experiments with statistical analysis were repeated and included multiple technical and biological replicates. The group sizes were determined based on prior immunological and virological studies of alphavirus infection in mice (PMID: 38483968, 32102875, 27452455, and 24131709). |
| Data exclusions | No data were excluded from the analysis. |
| Replication | All experiments included replicates and this information is found in the figure legends. |
| Randomization | Individual animals were randomly assigned to experimental groups. |
| Blinding | Researchers were not blinded. Many of the studies were analyzed independently by different members of the research team. |

# Reporting for specific materials, systems and methods

We require information from authors about some types of materials, experimental systems and methods used in many studies. Here, indicate whether each material, system or method listed is relevant to your study. If you are not sure if a list item applies to your research, read the appropriate section before selecting a response.

## Materials & experimental systems

| n/a | Involved in the study | |
|---|---|---|
| | ☒ | Antibodies |
| | ☒ | Eukaryotic cell lines |
| ☒ | | Palaeontology and archaeology |
| | ☒ | Animals and other organisms |
| ☒ | | Clinical data |
| ☒ | | Dual use research of concern |
| ☒ | | Plants |

## Methods

| n/a | Involved in the study | |
|---|---|---|
| ☒ | | ChIP-seq |
| | ☒ | Flow cytometry |
| ☒ | | MRI-based neuroimaging |

# Antibodies

Antibodies used

Biolegend Antibodies:
1. CD45 BUV785 Clone:30-F11 Catalog# 103149 Lot# 4276899
2. F4/80 BV605 Clone: BM8 Catalog# 123133 Lot# B426303
3. CD8 BV785 Clone: 53-6.7 Catalog# 100750 Lot# B403513
4. CD11b PerCpCy5.5 Clone: M1-70 Catalog# 101228 Lot# B410567
5. CD4 PECy7 Clone: GK1.5 Catalog# 100421 Lot# B399189
6. CD11c APC Clone: N418 Catalog # 117309 Lot# B396301
7. Ly6g APCCy7 Clone:1A8 Catalog# 127624 Lot# B425079
8. CD3 APC Clone:145-2C11 Catalog# 100311 Lot# B153087
9. CD11b FITC Clone: M1-70 Catalog# 101205 Lot# B430053
10. Ly6G PE Clone: 1A8 Catalog# 127607 Lot# B179814
11. NK1.1 PE Clone:PK136 Catalog# 108707 Lot# B320695
12. B220 PE Clone:RA3-6B2 Catalog# 103207 Lot# B375707
13. CD8 PE Clone:53-6.7 Catalog# 100707 Lot# B151200
14. CD90.2 APCCy7 Clone:30-H12 Catalog# 105327 Lot# B393093
15. F4/80 PE Clone: BM8 Catalog# 123109 Lot# B357449
16. IFNg PECy7 Clone:XMG12 Catalog# 505825 Lot# B251188

BD Biosciences:
1. CD45 BUV395 Clone:30-F11 Catalog# 564279 Lot# 4276899
2. MHCII BUV496 Clone: M5/114.15.2 Catalog# 750281 Lot# 5030619
3. TCRb BUV737 Clone: H57-697 Catalog# 612821 Lot# 4170404

eBioscience:
1. MHCII PE Clone: M5/114.15.2 Catalog# 12-5321-81 Lot# E025083

Invitrogen:
1. Live/Dead Stain Catalog# L34965 Lot# 2441360

BioXcell:
1. Isotype control: LTF-2
2. Anti-CD4: GK1.5
3. Anti-MHC-II: M5/114

Validation

The commercially obtained antibodies came with lot analyses and validation statements. The antibodies were further verified by flow cytometry.

# Eukaryotic cell lines

Policy information about cell lines and Sex and Gender in Research

Cell line source(s)

BHK-21 cells (ATCC CCL10) were obtained directly from ATCC.

Authentication

ATCC authenticates cell lines.

Mycoplasma contamination

BHK-21 cells used in these studies tested negative for mycoplasma.

Commonly misidentified lines
(See ICLAC register)

N/A

## Animals and other research organisms

Policy information about studies involving animals; ARRIVE guidelines recommended for reporting animal research, and Sex and Gender in Research

| | |
|---|---|
| Laboratory animals | Wild type C57BL/6J mice were obtained directly from The Jackson Laboratory (cat # 000664). Female and male mice (4-week-old) were used in all studies. |
| Wild animals | N/A |
| Reporting on sex | Female and male mice were used in this study, and the sex of the animals is reported in the manuscript. |
| Field-collected samples | N/A |
| Ethics oversight | This study was conducted in accordance with the recommendations in the Guide for the Care and Use of Laboratory Animals and the American Veterinary Medical Association (AVMA) Guidelines for the Euthanasia of Animals. All animal experiments conducted at the University of Colorado Anschutz Medical Campus were performed with the approval of the Institutional Animal Care and Use Committee (IACUC) of the University of Colorado School of Medicine (Assurance Number: A3269-01) under protocols 00026 and 00215. |

Note that full information on the approval of the study protocol must also be provided in the manuscript.

## Plants

| | |
|---|---|
| Seed stocks | N/A |
| Novel plant genotypes | N/A |
| Authentication | N/A |

## Flow Cytometry

### Plots

Confirm that:

☐ The axis labels state the marker and fluorochrome used (e.g. CD4-FITC).

☐ The axis scales are clearly visible. Include numbers along axes only for bottom left plot of group (a 'group' is an analysis of identical markers).

☒ All plots are contour plots with outliers or pseudocolor plots.

☒ A numerical value for number of cells or percentage (with statistics) is provided.

### Methodology

| | |
|---|---|
| Sample preparation | Single cell suspensions were generated from joint-associated tissues by mechanical and enzymatic digestion. Joint-associated tissues were horizontally agitated in RPMI 1640 (Gibco) medium supplemented with 10% fetal bovine serum (FBS), 100 U penicillin ml-1, and 100 g streptomycin ml-1 containing five 10 mm glass beads in a 15 mL conical for 2 h at 37°C. After incubation, digested tissues were filtered by passing through a 70 µm cell strainer. Single cell suspensions were blocked with anti-FcγRIII/II (2.4G2; BD Pharmingen) for 10 min at room temperature, stained with LIVE/DEAD Fixable Violet Dead Cell Stain (ThermoFisher) according to product instructions, then stained with the indicated antibodies for 45 min on ice. Cells were washed 2 times with FACS buffer (1% FBS, 2 µM EDTA, 20 mM HEPES in 1x PBS) and fixed by addition of PFA to a final concentration of 1% for 10 min at room temperature. For intracellular stains, fixed cells were incubated with the indicated antibodies in 0.1% saponin in FACS buffer for 0.5-2 h at room temperature, washed 3 times with 0.1% saponin in FACS buffer, and resuspended in 400 uLFACS buffer. |
| Instrument | Cells were analyzed using an Aurora (Cytek) flow cytometer. Cell sorting was performed using an XDP Flex instrument (Beckman Coulter). |
| Software | Data collection was performed using SpectroFlo software on the Aurora (Cytek) and Summit sofware on the XDP Flex (Beckman Coulter). Data was analyzed using FlowJo software v10.10 (Treestar) |
| Cell population abundance | Cell populations were determined after gating shown in Supplementary Figure 1 and Supplementary Figure 3. Post-sort flow cytometry analysis was used to determine the purity of sorted cell populations. |

| | |
|---|---|
| Gating strategy | A detailed description of the gating strategy is shown in Supplementary Figures 1 and 3. This includes FSC/SSC gates, singlets, live/dead, leukocytes (CD45+), dump gates, and all subsequent populations based on expression of specific markers. |

☒ Tick this box to confirm that a figure exemplifying the gating strategy is provided in the Supplementary Information.
